# Engineered mitochondria exert potent antitumor immunity as a cancer vaccine platform

**DOI:** 10.1038/s41423-024-01203-4

**Published:** 2024-08-20

**Authors:** Jingwen Luo, Fei Mo, Zhe Zhang, Weiqi Hong, Tianxia Lan, Yuan Cheng, Chunju Fang, Zhenfei Bi, Furong Qin, Jingyun Yang, Ziqi Zhang, Xue Li, Haiying Que, Jiayu Wang, Siyuan Chen, Yiming Wu, Li Yang, Jiong Li, Wei Wang, Chong Chen, Xiawei Wei

**Affiliations:** 1 Laboratory of Aging Research and Cancer Drug Target, State Key Laboratory of Biotherapy and Cancer Center, National Clinical Research Center for Geriatrics, West China Hospital, Sichuan University, Chengdu, Sichuan China; 2 Department of Medical Oncology, The First Affiliated Hospital of Kunming Medical University, Kunming, Yunnan China; 3 Zhejiang Provincial Key Laboratory of Pancreatic Disease, the First Affiliated Hospital, School of Medicine, Zhejiang University, Hangzhou, Zhejiang China

**Keywords:** Mitochondria vaccine, Antitumor immunity, TLR2, Cardiolipin, Tumour vaccines, Tumour vaccines

## Abstract

The preferable antigen delivery profile accompanied by sufficient adjuvants favors vaccine efficiency. Mitochondria, which feature prokaryotic characteristics and contain various damage-associated molecular patterns (DAMPs), are easily taken up by phagocytes and simultaneously activate innate immunity. In the current study, we established a mitochondria engineering platform for generating antigen-enriched mitochondria as cancer vaccine. Ovalbumin (OVA) and tyrosinase-related protein 2 (TRP2) were used as model antigens to synthesize fusion proteins with mitochondria-localized signal peptides. The lentiviral infection system was then employed to produce mitochondrial vaccines containing either OVA or TRP2. Engineered OVA- and TRP2-containing mitochondria (OVA-MITO and TRP2-MITO) were extracted and evaluated as potential cancer vaccines. Impressively, the engineered mitochondria vaccine demonstrated efficient antitumor effects when used as both prophylactic and therapeutic vaccines in murine tumor models. Mechanistically, OVA-MITO and TRP2-MITO potently recruited and activated dendritic cells (DCs) and induced a tumor-specific cell-mediated immunity. Moreover, DC activation by mitochondria vaccine critically involves TLR2 pathway and its lipid agonist, namely, cardiolipin derived from the mitochondrial membrane. The results demonstrated that engineered mitochondria are natively well-orchestrated carriers full of immune stimulants for antigen delivery, which could preferably target local dendritic cells and exert strong adaptive cellular immunity. This proof-of-concept study established a universal platform for vaccine construction with engineered mitochondria bearing alterable antigens for cancers as well as other diseases.

The concept of tumor vaccine aims to invoke antitumor adaptive immune responses to detect and eliminate tumors [1]. To date, there are a variety of approaches for therapeutic tumor vaccine development, including vaccines based on peptides, whole cells, nucleic acids, virus vectors, etc [2–5]. Nevertheless, most tumor vaccine candidates have failed to make clinical advancements in cancer therapy to date [6]. For example, the lack of clinical benefit of peptide-based tumor vaccines may be explained by inaccurate antigen delivery to nonprofessional antigen presenting cells (APCs) /noninflamed lymphoid organs [7]. Several pivotal trials using autologous tumor cells failed to reach their primary endpoints due to the lack of potent adjuvants to reverse immune suppression [8, 9]. Moreover, virus vector vaccines such as PROSTVAC and PANVAC did not generate sufficient immune responses as a single agent, which is now being tested in combination with checkpoint inhibitors in clinical trials [10, 11]. Based on these clinical experiences, efficient delivery of antigens to professional APCs and optimal adjuvants are key to combating the intrinsic tumor suppressive microenvironments and inducing adaptive immunity in tumor vaccine evaluation.

Recent research has demonstrated that the activation of pattern recognition receptors (PPRs) is important for generating potent and durable antitumor immunity for tumor vaccines [12, 13]. For instance, Toll-like receptor (TLR) agonists, while used as adjuvants, can indicate to the immune system that vaccine antigens are foreign and dangerous as damage-associated molecular patterns (DAMPs), which is especially important for peptide/protein vaccine platforms [14, 15]. Our previous work revealed the participation of DAMPs, such as oxidized mitochondrial DNA, as well as the STING pathway in the induction of antitumor immunity by irradiated cancer cell vaccines [16]. Several preclinical studies have suggested that agonists of TLR7/8 and TLR9 might synergistically contribute to the therapeutic effect of in situ tumor vaccines when used as adjuvants [17, 18].

Mitochondria are special organelles with prokaryotic characteristics that can potently activate immunity [19]. Many mitochondria-derived components have been recognized as DAMPs that stimulate PRRs, such as mtDNA for TLR9/STING [20]. Somatic mtDNA mutations have been found in some tumors, and the resulting altered mitochondrial proteins are potentially immunogenic and able to induce antitumor immune response [21]. In our previous research, it was observed that mitochondria isolated from cancer cells could be easily engulfed by dendritic cells (DCs), which are a natively well-orchestrated carrier full of immune stimulants preferably targeting local phagocytes, including APCs [16, 22]. The cytoplasmically synthesized precursor of the mitochondrial matrix enzyme, ornithine transcarbamylase (OTC), is anchored to mitochondria by its NH_2_-terminal leader peptide [23]. Thus, it is conceivable that the cytoplasmically expressed exogenous or endogenous antigen could be imported into mitochondria by the mitochondrial localization signal peptide from ornithine transcarbamylase (OTC leader sequence), which might act as DCs targeted delivery system with adequate source of natural adjuvants. In the current study, we aim to establish a platform to generate engineered mitochondria enriched with antigens by utilizing lentiviral vectors encoding antigen sequence and OTC leader sequence. The antigen-enriched mitochondria are isolated and prepared as a tumor vaccine, and the prophylactic and therapeutic effects of engineered mitochondria tumor vaccines were systemically evaluated in animal models.

## Result

### Construction of vaccine platform with model antigen-enriched mitochondria

**Fig. 1:**
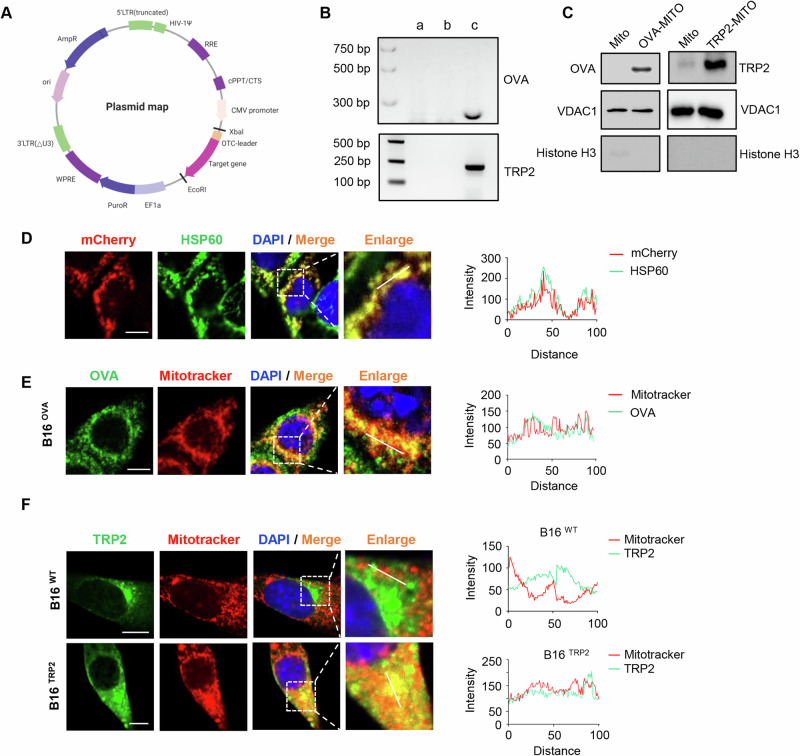
Construction of stable vaccine generation platform with engineered mitochondria bearing model antigen. **A** Schematic representation of the antigen expression plasmid containing the mitochondrial localization signal peptide sequence from ornithine transcarbamylase (OTC) and selected length of model antigen (OVA or TRP2) sequence. **B** Agarose gel electrophoresis showing mRNA expression levels of OVA or TRP2 in B16-F10 cells. a, untreated B16-F10 cells; b, B16-F10 cells transfected with null plasmid; c, constructed stable B16-F10 cell lines with mitochondria bearing model antigen (OVA or TRP2). **C** Immunoblot analysis of OVA or TRP2 levels in the mitochondria of B16-F10 cells or constructed stable B16-F10 cell lines with mitochondria bearing model antigen (OVA or TRP2). Anti-VDAC1 was used as a loading control. **D** Immunofluorescent analysis of the colocalization of exogenous mCherry with HSP60 in B16-F10 cells transfected with mCherry fluorescent protein expression plasmid with an OTC leader sequence. The fluorescence intensity corresponding to mCherry and HSP60 is shown on the right side. **E** Immunofluorescence analysis of the colocalization of exogenous OVA with MitoTracker in stable B16-F10 cell line with mitochondria bearing OVA (B16^OVA^). The fluorescence intensity corresponding to OVA and MitoTracker is shown on the right side. **F** Immunofluorescent analysis of the colocalization of exogenous TRP2 with MitoTracker in B16-F10 cells and stable B16-F10 cell line with mitochondria bearing TRP2 (B16^TRP2^). The fluorescence intensity corresponding to TRP2 and MitoTracker is shown on the right side. Scale bars represent 10 μm in (**D**–**F**)

Vaccine generation platform was established in the current study to obtain the antigen-enriched engineered mitochondria. We utilized a plasmid with mitochondrial localization signal peptide sequence and lentiviral infection system to construct a stable cell line with antigen contained mitochondria. Ovalbumin (OVA) or tyrosinase-related protein 2 (TRP2) was used as a model antigen and mitochondria-directed antigen plasmid (OTC-OVA and OTC-TRP2) containing both the OTC leader sequence and a selected length of the antigen sequence was constructed (Fig. 1A). Subsequently, B16-F10 cell lines stably expressing model antigen (OVA or TRP2) were obtained by lentiviral vector infection and puromycin selection. To determine whether the model antigen (OVA or TRP2) was successfully enriched in mitochondria by the OTC leader sequence in the selected cell lines, we first designed primers based on the OTC-OVA/TRP2 gene sequence and amplified the fragment through qPCR. Then, we checked the qPCR products by gel electrophoresis. The results demonstrated the OVA/TRP2 mRNA level in mitochondria (Fig. 1B). In accordance with the mRNA expression, the protein level of OVA or TRP2 in isolated mitochondria was also detected by western blot (Fig. 1C).

To confirm whether the exogenous antigen could be expressed and directed to mitochondria by the current platform, we constructed a mCherry fluorescent protein-expressing B16-F10 stable cell line to precisely observe the fluorescence location within the cell. We found that red fluorescence (mCherry) can be well merged with green fluorescence of mitochondria stained with HSP60 antibody, suggesting that the current platform for stable cell line screening could effectively generate exogenous antigen-containing mitochondria (Fig. 1D).

In the next step, to further observe the model antigen (OVA or TRP2) location in the constructed B16^OVA^ and B16^TRP2^ cell lines, immunofluorescence staining was performed. In the constructed stable cell line with OVA-contained mitochondria (B16^OVA^), the colocalization of OVA (green) and MitoTracker (red) was detected, as shown in Fig. 1E. In regard to the B16^TRP2^ cell line, although unmodified B16-F10 cells naturally expressed TRP2 throughout the cell (Fig. 1F), after the importation of antigen to mitochondria by OTC leader sequence, the colocalization of TRP2 and MitoTracker increased significantly (Fig. 1F). Together, these data demonstrate that we successfully constructed a stable vaccine generation platform with OVA- or TRP2-containing mitochondria. The mitochondria from B16^OVA^ and B16^TRP2^ cells were extracted as previously reported and prepared into a tumor vaccine for further experiments.

### Engineered mitochondria vaccine inhibits tumor growth in mice

**Fig. 2:**
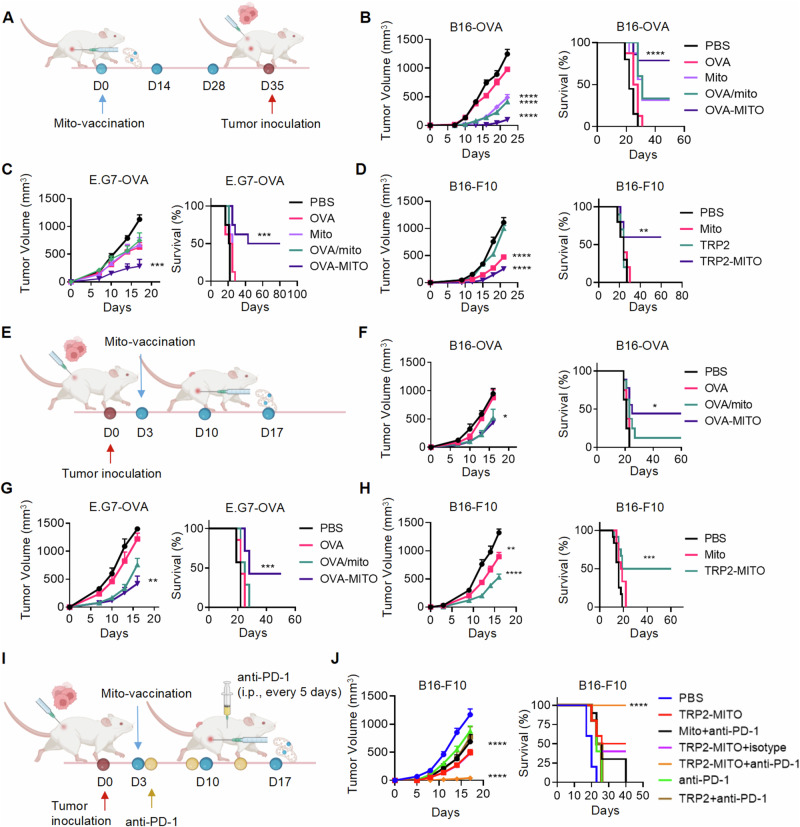
Engineered mitochondria vaccine inhibits tumor growth in mice. **A** Schematic representation of the prophylactic tumor vaccines (OVA-MITO or TRP2-MITO) for immunotherapy in mice. B16-OVA (**B**) and E.G7-OVA (**C**)-derived tumor-bearing mice received the indicated prophylactic treatments (5 μg soluble OVA, 50 μg Mito, 50 μg Mito plus 5 μg soluble OVA, or 50 μg OVA-MITO on day 0, 14 and 28). Mito represents the control mitochondria without tumor antigen. Tumor volumes and mouse survival measured at the indicated time points are shown. (*n* = 12 in (**B**) and *n* = 8 in **C**). **D**, B16-F10-derived tumor-bearing mice received the indicated prophylactic treatments (5 μg soluble TRP2, 50 μg Mito or 50 μg TRP2-MITO on day 0, 14 and 28). Tumor volumes and mouse survival measured at the indicated time points are shown (*n* = 10). **E** Schematic representation of the therapeutic tumor vaccines (OVA-MITO or TRP2-MITO) for immunotherapy in mice. B16-OVA (**F**) and E.G7-OVA (**G**)-derived tumor-bearing mice received the indicated therapeutic treatments (5 μg soluble OVA, 50 μg Mito plus 5 μg soluble OVA or 50 μg OVA-MITO on day 3, 10 and 17). Tumor volumes and mouse survival measured at the indicated time points are shown. (*n* = 8 in (**F**) and *n* = 7 in (**G**). **H** B16-F10-derived tumor-bearing mice received the indicated therapeutic treatments (50 μg Mito or 50 μg TRP2-MITO on day 3, 10 and 17). Tumor volumes and mouse survival measured at the indicated time points are shown. (*n* = 12). **I** Schematic representation of TRP2-MITO vaccines in combination with anti-PD-1 therapy in a murine B16-F10 tumor model. **J** B16-F10-derived tumor-bearing mice received the indicated therapeutic treatments (5 μg soluble TRP2, 50 μg TRP2-MITO or 50 μg Mito on day 3, 10, and 17, 10 mg/kg anti-PD-1 antibodies (Abs), or isotype-matched control antibody every 5 days). Tumor volumes and mouse survival measured at the indicated time points are shown. (*n* = 8). Data are presented as the mean values ± SEMs. One-way ANOVA was conducted for the analysis of tumor volumes, and the log-rank (Mantel-Cox) test was used for survival; *P < 0.05, **P < 0.01, ***P < 0.001, and ****P < 0.0001. See also Supplementary Fig. 1

The mitochondria were extracted previously reported [24]. Our antigen-containing mitochondria from the constructed antigen-expressing cell line were also extracted by the same protocols. The mitochondria of normal B16-F10 cells were also isolated and used as a control. OVA-containing mitochondria (named OVA-MITO) and TRP2-containing mitochondria (named TRP2-MITO) were characterized as tumor vaccines in mice. To evaluate the prophylactic efficacy of the tumor vaccine, mice were immunized with OVA-MITO or TRP2-MITO as the treatment regimen illustrated in Fig. 2A. OVA-MITO treatment as a prophylactic tumor vaccine significantly inhibited tumor growth and prolonged mouse survival compared with other groups in B16-OVA and E.G7-OVA murine subcutaneous tumor models (Fig. 2B, C). Vaccination with mitochondria alone or with the mixture of mitochondria and OVA had a partial therapeutic effect in the tumor models (Fig. 2B, C, right). However, compared to those in the control group, a better antitumor effect was achieved for the mice receiving OVA-MITO. In addition, the prophylactic effect of TRP2-MITO vaccine was also demonstrated by remarkably suppressed tumor growth and prolonged mouse survival in the B16-F10 model compared with the other groups (Fig. 2D).

In the next set of experiments, we tested the therapeutic efficacy of OVA-MITO and TRP2-MITO, with the injection regimen illustrated in Fig. 2E. After tumor inoculation, the three tumor vaccine treatments in the opposite flank also exhibited considerable therapeutic effects on different tumor models, as well as prolonged mouse survival compared with the other treatments (Fig. 2F–H). Moreover, We have conducted in vivo and in vitro experiments using cryo-mitochondrial and compared them with fresh mitochondrial vaccines. Our findings indicate a significant decrease in tumor growth inhibition in the cryo-mitochondrial vaccine group compared to the fresh mitochondrial vaccine group (Fig. 5K) (Supplementary information, Fig. S1B**)**. Additionally, the efficacy of cryo-mitochondrial vaccines was found to be comparatively lower in the in vitro experiment (Supplementary information, Fig. S1A). There is evidence that cancer vaccines could be combined with immune checkpoint blockade therapy, such as anti-PD-1 antibodies, to achieve better outcomes [25, 26]. In the current study, we examined the therapeutic efficacy of TRP2-mito vaccines in combination with anti-PD-1 therapy against a murine B16-F10 tumor model (Fig. 2I). Surprisingly, combination treatment with TRP2-mito and anti-PD-1 antibody led to complete inhibition of tumor growth and remarkably extended the survival of all mice in the treatment group (Fig. 2J). To investigate the therapeutic efficacy of a mitochondrial vaccine by examining the potential Antitumor effects of a mixture of antigens/mitochondria derived from non-tumorigenic cell lines or tissues. We conducted the following experiments to explore whether the mixture of antigens/mitochondria of non-tumorigenic cell lines or tissues has the same therapeutic effect as the mitochondrial vaccine we constructed. Our findings indicate that the tested mixture did not demonstrate significant Antitumor effects (Supplementary information, Fig. S1C, D). In conclusion, these results demonstrated that antigen-containing mitochondria (OVA-MITO and TRP2-MITO) exhibited potent Antitumor efficacy as prophylactic and therapeutic cancer treatment vaccines. Our findings suggest that the heightened immune response is contingent upon the mitochondrial phenotype of the host cell expressing the antigenic protein and the length of time the mitochondria are preserved. This phenomenon may be attributed to the enhanced ability of fresh and tumor cell mitochondria to elevate the steady-state levels of antigenic protein.

### Engineered mitochondria vaccine could effectively recruit dendritic cells to the injection site and enhance their maturation and function

**Fig. 3:**
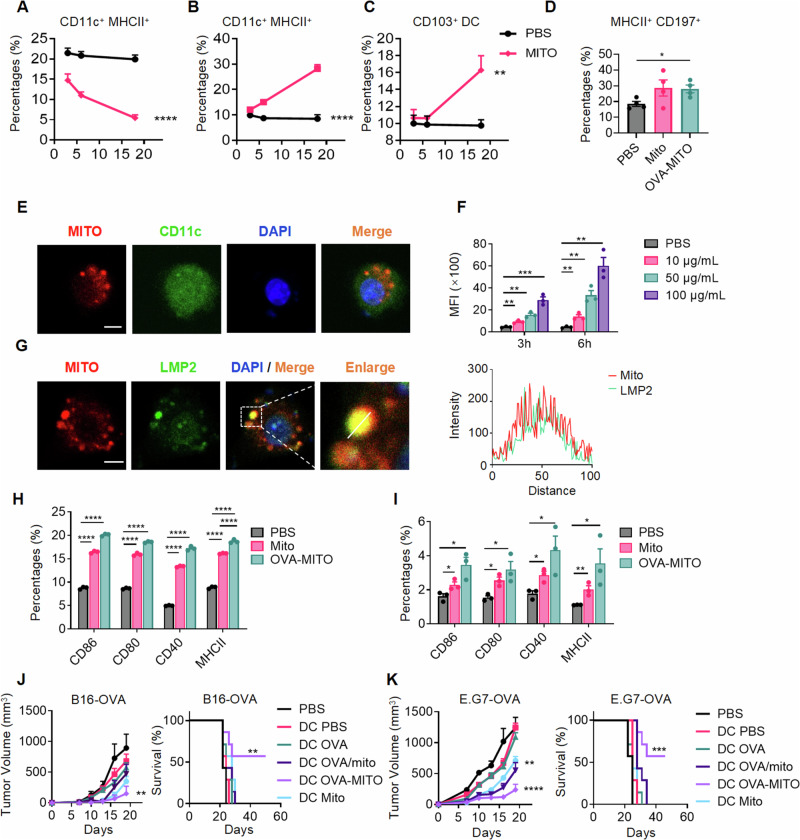
Engineered mitochondria vaccine could effectively recruit Dendritic cells and enhance their maturation and local antitumor immune response. **A** Flow cytometry analysis of CD11c^+^ MHC II^+^ DC accumulation at the injection site of mice after the subcutaneous injection (50 µg Mito, or PBS for 3 h, 6 h, and 18 h). (*n* = 5). Flow cytometry analysis of the number of CD11c^+^ MHC II^+^ DCs (**B**) and CD103^+^ DCs (**C**) migrating to the draining lymph nodes (dLNs) of mice after the subcutaneous injection (50 µg Mito, or PBS for 3 h, 6 h, and 18 h). (*n* = 5). **D** Flow cytometric analysis of CCR7 (CD197^+^) expression in MHC II^+^ DCs in the draining lymph nodes of mice after the subcutaneous injection (50 µg Mito alone, 50 µg OVA-MITO, or PBS for 18 h). (*n* = 4). **E** Immunofluorescence analysis of CD11c^+^ BMDCs incubated with mCherry-MITO (10 μg/mL) for 6 h. **F** Flow cytometric analysis of CD11c^+^ BMDCs treated with the indicated concentrations (PBS, 10 µg/mL, 50 µg/mL, or 100 µg/mL Mito) of MitoTracker™ Red CMXRos-stained mitochondria for 3 or 6 h. **G** Immunofluorescence analysis of the colocalization of exogenous mCherry-MITO with LMP2 in BMDCs treated with mCherry-MITO (10 μg/mL) for 3 h. The fluorescence intensity corresponding to mCherry and LMP2 is shown on the right side. **H** Flow cytometric analysis of CD86, CD80, CD40, and MHC II expression in BMDCs treated with Mito or OVA-MITO (10 μg/ml) in vitro for 24 h, the experiment was repeated twice. **I** Flow cytometric analysis of CD86, CD80, CD40, and MHC II expression in BMDCs in the draining lymph nodes of mice after the indicated treatments (50 μg Mito or 50 μg OVA-MITO into the hind footpad of mice for 18 h). (*n* = 3) The experiment was repeated twice. B16-OVA (**J**) and E.G7-OVA (**K**) tumor-bearing mice received the indicated therapeutic treatments (1 × 10^6^ DCs preincubated with PBS, OVA, OVA/mito, Mito and OVA-MITO at 10 μg/mL overnight, immunized on day 3, 10, and 17). Tumor volumes and mouse survival measured at the indicated time points are shown. (*n* = 7). Mito represents the control mitochondria without tumor antigen. Scale bars represent 10 μm in (**E**) and (**G**). Data are presented as the mean values ± SEMs. One-way ANOVA was conducted in (**A**–**D**), *t* test analysis was conducted in (**F**, **H**, and **I**), and log rank (Mantel-Cox) test was used for survival; *P < 0.05, **P < 0.01, ***P < 0.001, and ****P < 0.0001. See also Supplementary Fig. 2

To explore the underlying mechanism of mitochondria as an immunologic adjuvant, we evaluated how mitochondria stimulate dendritic cells (DCs). Dendritic cells are the major APCs responsible for antigen uptake and presentation and play critical roles in provoking adaptive antitumor immunity [27]. To explore whether the mitochondria vaccine could recruit DCs upon immunization, OVA-MITO or control mitochondria (isolated from B16-F10 cells) were subcutaneously injected into mice. Tissues from the injection site were isolated and analyzed with flow cytometry. After 18 h of starting MITO expression, a notable decrease in the overall amount of DCs in the skin was noted, along with an increase in migratory DCs in draining lymph nodes, indicating a quick departure of these cells from the skin (Fig. 3A, B). In addition, the proportions of dendritic cells migrating to draining lymph nodes increased with time, as well as CD103^+^ DCs in the draining lymph nodes (Fig. 3C). DCs are expected to take up antigens, traffic to lymph nodes, and present antigens via MHC class I or MHC class II. CD197 is a representative marker of DCs that reflects chemotaxis characteristics and lymph node homing ability [28]. The upregulation of CCR7 (CD197) expression in DCs in the draining lymph nodes in the mice immunized with OVA-MITO was also recorded (Fig. 3D).

To further confirm whether DCs could effectively uptake mitochondria-contained antigen, mCherry-MITO was added to isolated immature bone marrow-derived dendritic cells (BMDCs). The uptake of mCherry-MITO by BMDCs was observed by immunofluorescence staining of DCs with CD11c (Fig. 3E). BMDCs engulfed mitochondria in a dose- and time-dependent manner (Fig. 3F). Importantly, upon uptake by DCs, mCherry-MITO was found to be colocalized with DC proteasomes, as shown by staining for LMP2 (Fig. 3G), which indicates the crucial involvement of MHC class I presentation by DCs. The maturation of DCs is crucial for mounting a potent T-cell response. We found that after incubation with OVA-MITO in vitro, the expression levels of costimulatory markers (CD80, CD86, CD40) and MHC II were all significantly upregulated in DCs compared with the control groups (Fig. 3H). In addition, the upregulation of costimulatory markers and MHC II in DCs by OVA-MITO was also detected in vivo. After immunization with OVA-MITO, the DCs in the draining lymph nodes were effectively activated, as characterized by flow cytometry (Fig. 3I).

Based on the potent activation of OVA-MITO on dendritic cells, we further prepared an OVA-MITO-pulsed DC vaccine to validate whether the engineered mitochondria vaccine could be utilized in combination with a dendritic cell-based tumor vaccine. The therapeutic effects of the OVA-MITO-pulsed DC vaccine were studied in B16-OVA and E.G7-OVA subcutaneous tumor models. OVA-MITO-pulsed DC vaccine exhibited significant inhibitory effects on tumor growth and prolonged mouse survival compared with other treatment groups (Fig. 3J, K). Notably, the mixture of OVA/mitochondria-pulsed DC vaccine also showed partial effects on the E.G7-OVA tumor model; however, it was weaker than that of the OVA-MITO group. Moreover, the TRP2-MITO-pulsed DC vaccine also had strong antitumor ability toward the B16-F10 tumor model (Supplementary information, Fig. S2A). To conclude, these findings suggested that the engineered mitochondria vaccine could effectively recruit DCs to the injection site and induce DC maturation and migration to draining lymph nodes. OVA-MITO or TRP2-MITO could also be utilized in the form of a cell-based cancer vaccine and elicit potent therapeutic antitumor effects.

### Mitochondria vaccination activates T-cell immunity in the tumor microenvironment

**Fig. 4:**
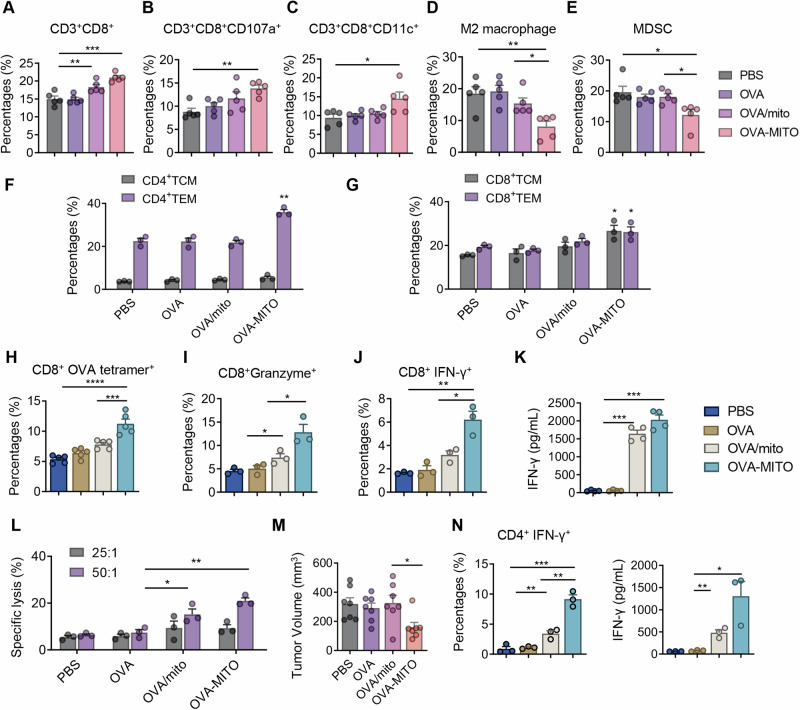
OVA-MITO vaccine activates T-cell immunity in the tumor microenvironment. Flow cytometric analysis of tumor-infiltrating CD3^+^ CD8^+^ T cell (**A**), CD3^+^ CD8^+^ CD107a^+^ T cell (**B**), and CD3^+^ CD8^+^ CD11c^+^ T cell (**C**) from mice preimmunized with the indicated treatments (5 μg soluble OVA, 50 μg Mito plus 5 μg soluble OVA or 50 μg OVA-MITO on day 0, 14 and 28). The data are illustrated as positive percentages in all tested cells. (*n* = 5). Flow cytometric analysis of tumor-infiltrating M2 macrophages (CD11b^+^, F4/80^+^, CD206^+^) (**D**), and MDSCs (CD11b^+^, Gr1^+^) (**E**) from mice preimmunized with the indicated treatments (5 μg soluble OVA, 50 μg Mito plus 5 μg soluble OVA or 50 μg OVA-MITO on day 0, 14 and 28). The data are illustrated as positive percentages in all tested cell. (*n* = 5). **F** Flow cytometric analysis of splenic CD4^+^ TCMs (CD44^+^CD62L^+^) and CD4^+^ TEMs (CD44^+^CD62L^low^) in mice receiving the indicated treatments (5 μg soluble OVA, 50 μg Mito plus 5 μg soluble OVA or 50 μg OVA-MITO on day 0, 14 and 28). The data are illustrated as positive percentages CD4^+^ cells (TCM, Central Memory T cell; TEM, Effector Memory T cell; *n* = 3). **G** Flow cytometric analysis of splenic CD8^+^ TCM cells (CD44^+^CD62L^+^) and CD8^+^ TEM cells (CD44^+^CD62L^low^) in mice receiving the indicated treatments (5 μg soluble OVA, 50 μg Mito plus 5 μg soluble OVA or 50 μg OVA-MITO on day 0, 14 and 28). The data are illustrated as positive percentages CD8^+^ cells (TCM, Central Memory T cell; TEM, Effector Memory T cell; *n* = 3). **H**–**J** Splenocytes from mice immunized the indicated treatment (5 μg soluble OVA, 50 μg Mito plus 5 μg soluble OVA or 50 μg OVA-MITO on day 0, 14 and 28) were isolated and subsequently cultured in vitro with OVA_257-264_ peptide for 72 h. Flow cytometric analysis of CD8^+^ OVA tetramer^+^ T cells (**H**), CD8^+^ Granzyme^+^ T cells (**I**), and CD8^+^ IFN-γ^+^ T cells (**J**). The data are illustrated as positive percentages in CD8^+^ cells (*n* = 3). **K** ELISA analysis of IFN-γ secretion in the supernatant of CD8^+^ T cells after the same treatment as (**H**–**J**). (*n* = 4). The experiment was repeated twice. **L** Splenic lymphocytes from mice that had received the indicated treatment (5 μg soluble OVA, 50 μg Mito plus 5 μg soluble OVA or 50 μg OVA-MITO on day 0, 14 and 28) were isolated and subsequently cultured with B16-OVA cells at different ratios (25:1, 50:1) for 6 h. The specific lysis analysis of B16-OVA cells after splenic T-cell stimulation. (*n* = 3). **M**, Splenic lymphocytes were isolated from mice received the indicated treatments (immunized with 5 μg soluble OVA, 50 μg mito plus 5 μg soluble OVA or 50 μg OVA-MITO in the prophylactic vaccine mode). A total of 1 × 10^7^ spleen lymphocytes prepared from immunized mice were injected 1 day before inoculation of B16-OVA cells. Tumor volumes measured at the indicated treatments are shown. (*n* = 7). **N**, Splenocytes from mice that had received the indicated treatment (5 μg soluble OVA, 50 μg Mito plus 5 μg soluble OVA or 50 μg OVA-MITO on day 0, 14 and 28) were isolated and subsequently cultured in vitro with OVA_323-339_ peptide for 72 h. Flow cytometric analysis of IFN-γ^+^ CD4^+^ Th1 cells (Left). The data are illustrated as positive percentages in CD4^+^ cells. ELISA analysis of IFN-γ secretion in the supernatant of Th1 CD4^+^ T cells (right). (*n* = 3). The experiment was repeated twice. Data are presented as the mean values ± SEMs. One-way ANOVA was conducted in (**A**–**N**); *P < 0.05, **P < 0.01, ***P < 0.001, and ****P < 0.0001. See also Supplementary Fig. 3

To further evaluate how the OVA-MITO vaccine stimulates an adaptive antitumor immune response, the tumor microenvironment, especially the T-cell population, was characterized. The effects of antitumor vaccines were reported to crucially depend on the activation of the cell-mediated immune response, such as the activation of cytotoxic T cells [29]. To determine the effects of the mitochondria vaccine on the activation of cellular immunity, mice were immunized three times with OVA-MITO, inoculated with tumors and sacrificed on Day 14 after B16-OVA cell inoculation. The population of immune cells, especially cytotoxic T cells, in tumors was analyzed by flow cytometry. We observed that OVA-MITO vaccination significantly increased the number of tumor-infiltrating CD8^+^ T cells (Fig. 4A). At the same time, the percentages of tumor-infiltrating CD8^+^ CD107a^+^ T cells and CD8^+^ CD11c^+^ T cells were both significantly elevated after OVA-MITO treatment (Fig. 4B, C). These markers were reported to be tightly associated with the tumoricidal property of CD8^+^ T cells [30, 31]. The activation of the local immune microenvironment was also supported by the decreased infiltration of M2 macrophages, as well as suppressive myeloid-derived suppressor cells (Fig. 4D, E). Moreover, we also characterized the proportions of activated memory T cells in the spleen in the treated mice. The OVA-MITO group exhibited efficiently increased percentages of CD4^+^ effector memory T cells and showed a slight increase in the percentages of both CD8^+^ central memory T cells and CD8^+^ effector memory T cells compared with the other groups (Fig. 4F, G).

To further explore whether the OVA-MITO/TRP2-MITO vaccine could induce antigen-specific cell-mediated adaptive immunity, splenocytes were isolated from mice immunized with OVA-MITO and subsequently stimulated with MHC-I-binding OVA_257-264_ (SIINFEKL) peptide. A significant increase in the percentages of OVA-specific CD8^+^ T cells in the OVA-MITO treatment group was detected with H-2Kb OVA tetramers. A slight elevation in the OVA-specific CD8^+^ T-cell population was also detected in the mixture of OVA and MITO (OVA/mito) treatment group compared with the control groups (Fig. 4H). Granzyme B is important for CD8^+^ T cells to kill their targets [32], and the population of Granzyme B-secreting CD8^+^ T cells was also increased after OVA-MITO vaccine treatment. (Fig. 4I). In addition, the number of IFN-γ-secreting CD8^+^ T cells and IFN-γ secretion in the supernatant were significantly elevated in the OVA-MITO and OVA/mito groups after incubation with OVA_257-264_ peptide (Fig. 4J, K). Moreover, the cytotoxicity analysis showed that the splenic lymphocytes from OVA-MITO-immunized mice, after stimulation, exhibited significant cytotoxicity for B16-OVA target cells compared with that from other groups (Fig. 4L). To further confirm its antitumor immunity, we performed an adoptive transfer experiment in C57BL/6 mice [33]. After receiving 3 doses of OVA-MITO injection, splenic lymphocytes were isolated and transferred to C57BL/6 mice to assess antitumor immunity. Tumor growth was significantly inhibited in the mice that received the lymphocytes from the OVA-MITO treatment group compared with those that received the cells from the other groups (Fig. 4M). Similar results were obtained after the characterization of TRP2-specific CD8^+^ T cells, granzyme B secretion, and IFN-γ production (Supplementary information, Fig. S3A–C). It was also observed that the CD4^+^ T cells might be involved in cytotoxic T-cell (CTL) production because OVA-MITO treatment notably increased the proportion of CD4^+^ IFN-γ-producing Th1 cells (Fig. 4N left), as well as the secretion of IFN-γ by CD4^+^ cells (Fig. 4N right). The results indicated that the systemic antitumor effects of engineered mitochondria vaccines might critically depend on the cell-mediated immune response via Th1 cells [34].

### Mitochondria vaccination promotes dendritic cell maturation by regulating the TLR2-mediated pathway

**Fig. 5:**
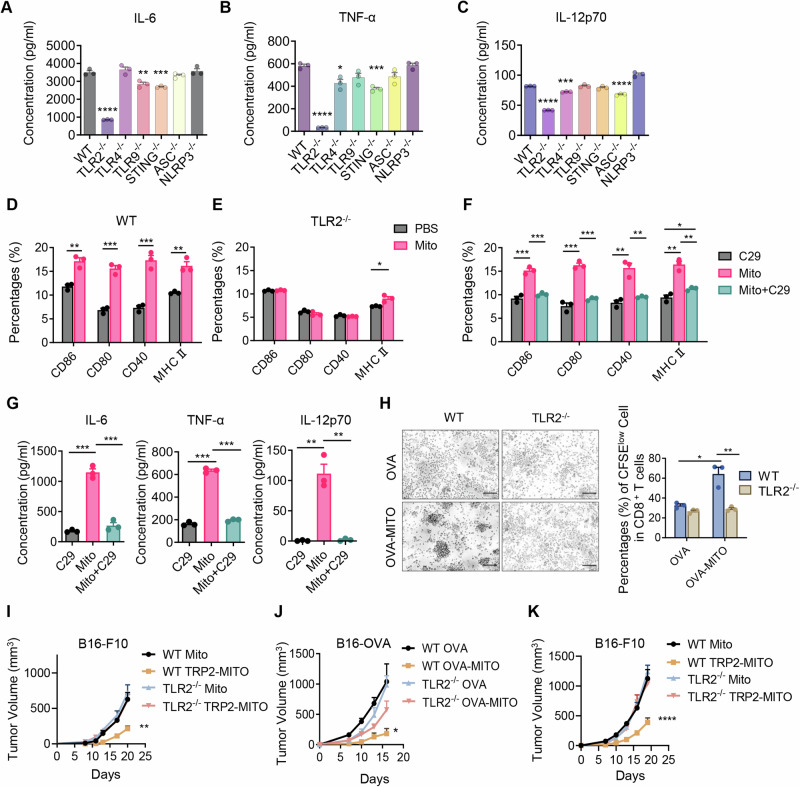
Mitochondria vaccines promote dendritic cell maturation by regulating the TLR2-mediated pathway. ELISA analysis of IL-6 (**A**), TNF-α (**B**), and IL-12p70 (**C**) levels in the supernatant of different knockout CD11c^+^ BMDCs that were incubated with mitochondria (10 μg/mL) for 24 h. Flow cytometric analysis of CD86, CD80, CD40, and MHC II in WT (**D**) and TLR2^−/−^ (**E**) CD11c^+^ BMDCs that were incubated with or without mitochondria (10 μg/mL) for 24 h. (*n* = 3). **F** Flow cytometric analysis of CD86, CD80, CD40, and MHC II of CD11c^+^ BMDCs (pretreatment of BMDCs with 100 Μm C29 with for 2 h, subsequently stimulated with or without 10 μg/mL mitochondria for 24 h). (*n* = 3). **G** ELISA analysis of IL-6, TNF-α and IL-12p70 levels in the supernatant of BMDCs that received the same treatments as (**F**) (*n* = 3). **H** BMDCs (2 × 10^5^) from WT or TLR2^−/−^ mice were preincubated with OVA (1 μg/mL) or OVA-MITO (10 μg/mL) and subsequently cocultured with CFSE-labeled CD8^+^ T cells (1 × 10^6^) from OT-I mice for 48 h. Phase contrast images of OT-I CD8^+^ T cells, Scale bars, 100 μm. The experiment was repeated twice. **I** B16-F10 tumor-bearing WT and TLR2^−/−^ mice received the indicated prophylactic treatments (50 μg Mito or 50 μg TRP2-MITO on day 0, 14, and 28). Tumor volumes measured at the indicated time points are shown. (*n* = 8). **J** B16-OVA tumor-bearing WT and TLR2^−/−^ mice received the indicated prophylactic treatments (5 μg soluble OVA or 50 μg OVA-MITO on day 0, 14 and 28). Tumor volumes measured at the indicated time points are shown (*n* = 8). **K** B16-F10 tumor-bearing WT and TLR2^−/−^ mice received the indicated therapeutic treatments on the opposite frank (50 μg Mito or 50 μg TRP2-MITO on day 3, 10 and 17). Tumor volumes measured at the indicated time points are shown. (*n* = 12 in the WT Mito group; *n* = 11 in the other groups). Data are presented as the mean values ± SEMs. One-way ANOVA was conducted in (**A**–**C**) and (**I**–**K**), and *t* test analysis was conducted in (**D**–**H)**; *P < 0.05, **P < 0.01, ***P < 0.001, and ****P < 0.0001. See also Supplementary Fig. 4

Mitochondria are an abundant source of damage-associated molecular patterns (DAMPs), which are potent stimulants for pattern recognition receptors (PRRs), such as Toll-like receptors (TLRs) [19, 20]. To further investigate how the engineered mitochondria vaccine activates the adaptive immune response, we utilized a series of knockout mice to examine the underlying mechanisms. The dendritic cells from different knockout mice were incubated with mitochondria, and the supernatants of DCs were analyzed by ELISA for the presence of IL-6, TNF-α, and IL-12p70 after 24 h of incubation (Fig. 5A–C). Surprisingly, the production of IL-6, TNF-α, and IL-12p70 was dramatically reduced in TLR2^−/−^ DCs. To further elucidate the role of TLR2 in DC activation, we analyzed costimulatory molecules, and MHC II expression on DCs from WT or TLR2^−/−^ mice, with or without mitochondria (10 µg/ml) stimulation. The elevated expression of CD80, CD86, CD40 and MHC II was detected in DCs from WT after mitochondria stimulation, which was diminished while using DCs from TLR2^−/−^ mice (Fig. 5D, E). Consistent with the finding, pretreatment with TLR2-specific inhibitor C29 significantly downregulated the expression level of the maturation markers and the secretion of IL-6, TNF-α, and IL-12p70 by DCs in the culture supernatants in comparison with the controls (Fig. 5F, G). These results suggested that mitochondria stimulate DCs to secrete pro-inflammatory cytokines and upregulate costimulatory molecule expression via the TLR2 pathway, thereby potently activating T cells.

To further characterize the role of TLR2 in antigen presentation for the mitochondria vaccine, BMDCs from wild-type or TLR2^−/−^ mice were preincubated with OVA or OVA-MITO and subsequently cocultured with CFSE-labeled CD8^+^ T cells from OT-I mice [35]. The results showed that wild-type BMDCs incubated with OVA-MITO could significantly promote the proliferation of OT-I CD8^+^ T cells and form evident cell clusters. In contrast, a significant reduction in T-cell proliferation was recorded after the incubation of OVA-MITO-pulsed TLR2^−/−^ BMDCs with OT-I CD8^+^ T cells. (Fig. 5H). To further confirm the role of TLR2 in immune activation, we next studied whether the engineered mitochondria vaccine could stimulate the cellular immune response in TLR2^−/−^ mice. The results showed that vaccination with OVA-MITO led to effective induction of OVA-specific CD8^+^ T cells in WT mice; however, this effect was not detected in the TLR2^−/−^ group (Supplementary information, Fig. S4A). There was also no increase in neither the percentages of IFN-γ-secreting CD4^+^ and CD8^+^ T cells nor in the concentration of IFN-γ in the supernatant of splenocytes from TLR2^−/−^ mice after OVA-MITO/TRP2-MITO treatment (Supplementary information, Fig. S4B-C).

To determine the role of TLR2 in the antitumor effects of the engineered mitochondria vaccine, the prophylactic and therapeutic effects of mitochondria were compared in WT and TLR2^/-^ mice. The prophylactic effect of TRP2-MITO was validated in the B16-F10 tumor model, and TRP2-MITO did not exert an antitumorigenic effect when used to treat TLR2^−/−^ mice (Fig. 5I). A similar tendency was also shown in OVA-MITO. Knockout of TLR2 in mice dramatically diminished the tumor inhibitory effects of OVA-MITO as a prophylactic tumor vaccine (Fig. 5J). In addition, the efficacy of TRP2-MITO as a therapeutic vaccine was also significantly affected by TLR2 knockout in mice compared with that in the WT group (Fig. 5K). These results demonstrated the indispensable role of TLR2 in the activation of DCs and the cellular immune response induced by engineered mitochondria vaccines.

### Cardiolipin from mitochondria play a major role in dendritic cell maturation and antitumor immune response through TLR2

**Fig. 6:**
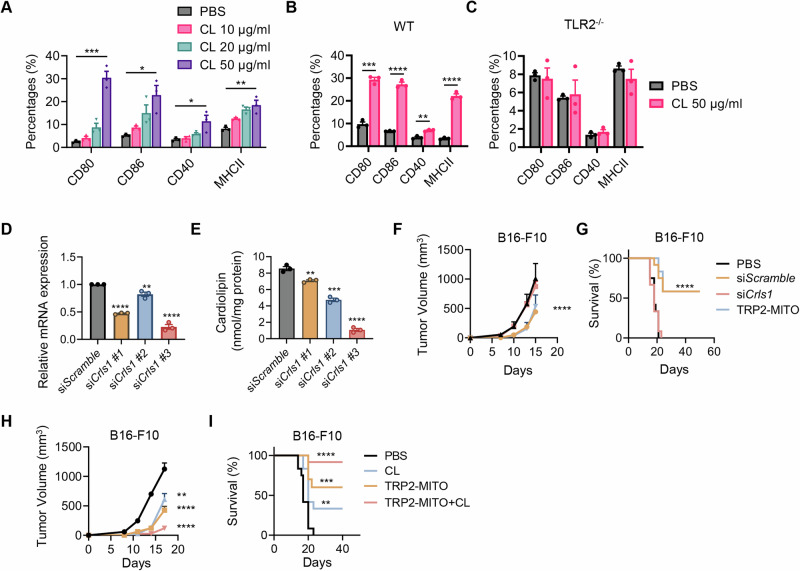
Cardiolipin from mitochondria play a major role in dendritic cell maturation and antitumor immune response through TLR2. **A** Flow cytometric analysis of CD86, CD80, CD40, and MHC II of CD11c^+^ BMDCs that were treated with the indicated concentrations (PBS, 10 μg/mL, 20 μg/mL or 50 μg/mL of cardiolipin for 24 h. CL is for cardiolipin (*n* = 3). The experiment was repeated twice. Flow cytometric analysis of CD86, CD80, CD40, and MHC II in WT (**B**) and TLR2^−/−^ (**C**) CD11c^+^ BMDCs that were treated with or without CL (50 μg/mL) for 24 h. (*n* = 3). The experiment was repeated twice. **D** qRT-PCR analysis of *Crls1* in B16-F10 cells transfected with si*Crls1* or si*Scramble* for 48 h. **E** Analysis of CL level in mitochondria in B16-F10 cells transfected with si*Crls1* or si*Scramble* for 48 h. **F-G** B16-F10-derived tumor-bearing mice received the indicated therapeutic treatments (50 μg TRP2-MITO, 50 μg CL-downregulated TRP2-MITO with si*Crls1* or with si*Scramble* on day 3, 10 and 17). Tumor volumes measured and mouse survival measured at the indicated time points are shown. (*n* = 12). **H**–**I** B16-F10-derived tumor-bearing mice received the indicated subcutaneous injection (50 μg TRP2-MITO on day 3, 10 and 5 mg/kg CL every 3 days). Tumor volumes and mouse survival measured at the indicated time points are shown (*n* = 12). Data are presented as the mean values ± SEMs. T test analysis was conducted in (**A–C**), one-way ANOVA was conducted in (**D**–**H**), and the log rank (Mantel-Cox) test was used for survival; **P < 0.01, ***P < 0.001, and ****P < 0.0001

Next, we focused on seeking potential TLR2 activators in mitochondria-derived DAMPs. Cardiolipin (CL) is a unique lipid component located in the inner mitochondrial membrane that has been reported to activate several pattern recognition receptors, including TLR2 [36]. In the next set of experiments, we studied whether CL plays a role in the immune-stimulating effect of the mitochondria vaccine. Coincubation of CLs with BMDCs significantly induced DC maturation and the upregulation of costimulatory molecule expression in a CL dose-dependent manner (Fig. 6A). Moreover, to elucidate whether the activation of DCs by CL relies critically on the TLR2 pathway, BMDCs from TLR2^−/−^ mice were also stimulated with CL. Interestingly, the expression levels of CD80, CD86, CD40, and MHC II in CL-stimulated TLR2^−/−^ BMDCs exhibited little change compared to those in the PBS group, while WT BMDCs could be effectively activated by CL (Fig. 6B, C).

To further explore whether CL is involved in the antitumor effects of the mitochondria vaccine, we knocked down CL synthase 1 (Crls1) with siRNAs to manipulate the content of CL in B16-F10 cells using a scrambled siRNA as a control [24]. The addition of si*Crls1* led to a significant CL reduction at the mRNA level (Fig. 6D). At 48 h post-transfection, the highest efficiency was observed by utilizing si*Crls1*#3, which reduced CL mRNA levels by 88% compared to parental cells (Fig. 6D). si*Crls1*#3 treatment in B16-F10 also caused a significant reduction in the CL lipid concentration in the extracted mitochondria as detected (Fig. 6E), which was used in the subsequent experiment.

In the next set of experiments, we treated B16-F10 tumor-bearing mice with CL-downregulated TRP2-MITO. The results strongly demonstrated the vital role of CL in the therapeutic efficacy of the mitochondria vaccine, as the knockdown of CL in TRP2-MITO resulted in significantly attenuated antitumor effects (Fig. 6F). Moreover, in accordance with our hypothesis, the addition of exogenous CL with TRP2-MITO further enhanced the therapeutic effect of TRP2-MITO and even prolonged mouse survival compared with the control groups (Fig. 6G). Taken together, these results provide direct evidence that CL from mitochondria is essential for promoting DC activation through the TLR2 pathway, provoking the antitumor immune response and is critically associated with the antitumor effects of the engineered mitochondria vaccine.

### Safety analysis of engineered mitochondria vaccines

To address the safety issue of the engineered mitochondria vaccine, mice that received three doses of OVA-MITO/TRP2-MITO were sacrificed, and blood samples were collected. No adverse events were detected, such as behavioral changes, ruffling fur, appetite loss, etc. No significant changes in body weight were recorded every day (Supplementary information, Fig. S5A–C). Additionally, after treatment with the OVA-MITO/TRP2-MITO vaccine, the serum biochemical parameters (ALT, AST, ALP, CREA, BUN, and UA) in the mice from either the control group or the experimental group showed no distinct abnormalities (Supplementary information, Fig. S5B–D). To further explore whether the mitochondria vaccine would produce the anti-mitochondrial antibody in primary organ tissues of mice, we performed anti-mitochondrial antibody detection assays. There were no differences in the major organs of mice in each experimental group compared to the control group (Fig. S5E). These results demonstrated the good safety profile of the engineered mitochondria vaccine in the current study.

## Discussion

In addition to powering cells, mitochondria play a crucial role in immunity [37–41]. Due to their close structural resemblance to bacteria, the release or exposure of their components upon cellular injury can initiate an innate immune response [42]. Numerous previous studies in mitochondrial biology have uncovered a spectrum of DAMPs from mitochondria, which include mitochondrial DNA (mtDNA), n-formyl peptide, and cardiolipin [42, 43]. These DAMPs serve as the foundation for initiation or enhancement of mitochondria-mediated innate immunity. The development of novel cancer immunotherapies around mitochondria is currently a hot topic of growing interest. A recent study has found that the oscillations in mitochondrial morphology, coupled with their associated metabolic output, significantly impact the ability of DCs to process antigens and present them to T cells [44]. Additionally, a mitochondria-targeted vaccine can induce a robust Antitumor immune response by activating tumor antigen-specific CD8^+^ T cells. This indicates that merging antigens with mitochondria could enhance antigen processing and holds promise for future vaccine design [38]. Here, our study demonstrates that mitochondria serve as immune response initiators and efficient carriers of antigens, thereby enhancing the immune response of antitumor vaccines.

Several observations concerning mitochondria, DCs, tumor vaccines, and the TLR2 pathway were made in the current study. We established a targeted antigen-expressing platform for generating engineered mitochondria bearing selective antigens. In the present study, OVA and TRP2, as model antigens, were imported into mitochondria by utilizing an OTC leader sequence-containing plasmid and lentiviral infection system. The success in the construction of a stable cell line with OVA- or TRP2-containing mitochondria was confirmed by RT-PCR, western blotting and immunofluorescence staining. Furthermore, ELISA technology was utilized for absolute quantification of OVA content in OVA-mito, revealing that 50 μg of OVA mito contained ~26.5 pg of OVA protein. Subsequent in vitro and in vivo experiments included a control group (OVA), with the dosage of OVA in the OVA/mito group significantly exceeding that in the OVA-mito group. The rationale for utilizing a comparatively elevated concentration of OVA protein as a control group is to illustrate the continued efficacy of the OVA-mito group in enhancing tumor-specific immunity even when exposed to minimal levels of antigens. The engineered mitochondria were isolated according to a previous protocol and evaluated as prophylactic and therapeutic tumor vaccines in animal models. OVA-MITO and TRP-MITO exhibited significant antitumor effects and prolonged mouse survival when used for both tumor prevention and tumor therapy. Moreover, combinational treatment with anti-PD1 therapy further improved the tumor suppressive efficiency. The critical roles of dendritic cells in the antitumor effect of the vaccines were fully addressed for the potent induction of DC activation and migration in dLNs by the engineered mitochondria. Accordingly, our results also revealed that adaptive cellular immunity was provoked by OVA-MITO/TRP-MITO vaccination with substantially increased percentages of activated cytotoxic T cells and effector memory T cells in the mouse spleen. We also revealed that the underlying mechanism for DC activation and antitumor immunity stimulation by OVA-MITO/TRP2-MITO involves the TLR2 pathway and its potent agonist-derived mitochondrial membrane, namely, cardiolipin. Taken together, we have proven that engineered mitochondria bearing antigens could exert potent antitumor cellular immunity as a platform for generating universal tumor vaccines, which could theoretically treat various types of tumors by loading different antigens.

In the current study, the engineered mitochondria were generated from the stable cell lines with antigen-enriched mitochondria. The methodology of the construction of a stable cell line utilizing a lentivirus system has been well established, widely used, and which is also preferably used in the industrial production of engineered mitochondria with large-scale cell culture. The stable cell line could theoretically enrich any antigen in mitochondria simply by altering the sequence of the lentivirus vector. Therefore, it is considered a universal platform for MITO-vaccine generation. Several studies reported that the phenomenon that mitochondria are easily taken up by dendritic cells [16, 21, 22]. Based on its preferable nature toward phagocytes, polymer-wrapped mitochondria have been prepared and have the potential to be used as a targeted delivery system for nucleic cargos to replace unhealthy mitochondria in tumor cells [24]. However, little is known about whether mitochondria are suitable for use as antigen delivery carriers in tumor vaccines. In fact, many of the mitochondrial compositions have been identified as immune-stimulating contents [19, 20]. Based on the symbiosis theory, mitochondria derived from prokaryotic ancestors possess the unique features of bacteria, such as the bilayer membrane, circular mtDNA with CpG repeats, synthesis of cardiolipin and formyl peptide [45, 46]. Mitochondria-derived DAMPs have been reported to activate the innate immune response under normal physiological conditions [19]. Therefore, compared with systemic drug delivery carriers, there is a greater potential for mitochondria to act as local antigen carriers with a strong adjuvant effect [47]. In this study, the OTC leader sequence was utilized to precisely locate antigens on mitochondria. The engineered mitochondria vaccine demonstrated preventive and therapeutic effects on tumor growth while effectively reversing the immunosuppressive tumor microenvironment. The preliminary experimental results showed that the simple mitochondrial group had a specific antitumor effect but was significantly weaker than the antigen-loaded mitochondrial vaccine. Besides, due to its low affinity, rapid degradation, and immunological toleration, TRP2 alone does not achieve antitumor or therapeutic effects without nanocarriers. The resistance of immune checkpoint inhibitors is mainly related to the following factors: severe depletion of T cells, tumor-specific CD8^+^T lymphocyte deficiency, Immunosuppression of tumor microenvironment, and immune deficiency in lymphocyte desert-type tumors [48]. Through the above experiments, we have verified that antigen-loaded mitochondrial vaccine can induce the production of CTL cells, increase the number of memory T cells, and improve the tumor microenvironment. Therefore, combining immune checkpoint inhibitors (anti-PD-1 antibodies) and antigen-loaded mitochondrial vaccines has a better synergistic effect than simple mitochondria with anti-PD-1 inhibitors or TRP2 combined with anti-PD-1 inhibitors. Encouragingly, we observed that the combination therapy of anti-PD-1 and antigen-loaded mitochondrial vaccine inhibited tumor growth and prolonged the survival of all mice in the treatment group.

Dendritic cell vaccines play an important role in the development of tumor vaccines [27]. The study observed that the mixture of antigens/mitochondria-pulsed DC vaccine also has some Antitumor effects. Still, there is no significant difference in efficacy compared to the DC TRP2/OVA group. According to previous research results, mitochondrial vaccines alone have weaker Antitumor effects than those loaded with antigens. Therefore, a mitochondria-pulsed DC vaccine was also found to be significantly effective in inhibiting tumor growth in this study. This can be attributed to the potent activation of DCs by antigen-containing mitochondria. Engineered mitochondria induced DC cell maturation both in vitro and in vivo. Subcutaneous immunization with mitochondria vaccines can effectively recruit DCs aggregates at local sites and activate DC migration to draining lymph nodes to promote antigen presentation. It has been reported that the unsatisfactory clinical results of the first generation of DC vaccines are largely attributed to the insufficient activation of DCs to present costimulatory factors and cytokines [27]. The roles of TLR agonist adjuvants for potent DC activation and generation of strong T-cell responses are critically addressed [49–52]. Although TLRs and their signaling pathways play important roles in DC maturation, it should also be noted that the types of TLRs expressed in human and mouse dendritic cells are different. For instance, human professional Ag-presenting DCs express only TLR2 and TLR3, while mouse DCs express TLR7 and TLR9 in addition to TLR2 family members (TLR1, 2, and 6) and TLR3 [53]. In this study, we focused on the indispensable role of TLR2 signaling in the function of OVA-MITO/TRP2-MITO. However, it cannot be ruled out that multiple pathways are involved, e.g., a slight difference in cytokine production was observed in TLR4^−/−^ and TLR9^−/−^ DCs after OVA-MITO incubation. Nevertheless, TLR2 knockout significantly diminished the inhibitory effects of OVA-MITO/TRP2-MITO on tumor growth in both the prophylactic model and therapeutic model. The addition of the TLR2 activator exogenous cardiolipin to the mitochondria vaccine could also virtually enhance DC activation and the therapeutic effect.

The development of cancer vaccines has benefited from the introduction of adjuvants that can trigger PRRs [54]. Recently, the commonly used approach for peptide-based tumor vaccines is the codelivery of peptide antigens and TLR agonists via nanotechnology-based delivery systems [14, 55]. Many of them demonstrated encouraging outcomes during the preclinical study; however, they would meet several obstacles in the translational stage, such as difficulty in large-scale production to maintain reproducibility and to achieve controllable pharmacokinetic behavior of both biomaterials and cargos. In the current study, the introduction of mitochondrial OTC leader sequences allowed us to establish stable cell lines with antigen enriched mitochondria. OVA and TRP2 were used as the models of endogenous and exogenous antigens to conduct the proof-of-concept study, and OVA/TRP2-containing mitochondria were produced from stable antigen-expressing cell lines with a simple isolation protocol. The distribution of OVA/TRP2 in cells was characterized by colocalization of HSP60 with MitoTracker as observed by immunofluorescence staining. Thus, engineered mitochondria are an easy-to-produce integral system containing antigens and abundant adjuvants for favorable delivery to DCs. Such an antigen-delivery platform based on engineered mitochondria might also be utilized to develop preventative vaccines for infectious diseases in the future.

This study still has some limitations. Firstly, the integrity of the mitochondria in the Mito-TRP2 vaccine could not be guaranteed even after extraction and purification. The potential impact of these residual mitochondrial fragments on the tumor immune response remains uncertain. In addition, tumor immune evasion could result from activation of TLR2-mediated signaling pathways. A previous study demonstrated that TLR2/10 heterodimers displayed inhibitory effects on human DCs activation [56]. Notably, TLR2 activation can also induce the accumulation of regulatory T cells in the tumor environment, thereby impairing vaccine efficacy [57]. In line with a paralleled study [58], we revealed that the combination of a mitochondrial vaccine with an anti-PD-L1 antibody further enhanced the efficacy of tumor immunotherapy.

Nevertheless, our study present massive objective evidence of mitochondrial-triggered innate immune activation via TLR2 signaling pathway. Mitochondrial vaccines exhibit ease of production, minimal side effects, and safety, thus justifying their further investigation in clinical trials. In the future, engineered mitochondria could serve as an antigen delivery platform for developing prophylactic vaccines against infectious diseases.

## Methods

### Cell culture

B16-F10 (ATCC CRL-6475) and B16-OVA cells (OVA-transfected B16-F10 cells) were cultured in DMEM (FBS, Gibco) supplemented with 10% fetal bovine serum (Gibco), penicillin (100 U/ml), and streptomycin (100 mg/ml). E.G7-OVA cells (ATCC CRL-2113) (OVA-transfected EL4 murine T lymphoma cells) were cultured in RPMI 1640 (Gibco) supplemented with 10% FBS (Gibco), penicillin (100 U/ml), streptomycin (100 mg/ml), and G418 (0.4 mg/ml) (Invivogen, ant-gn-1). Human embryonic kidney 293T (HEK293T) (ATCC CRL-11268) cells were cultured in DMEM (Gibco) complete medium. Mouse primary spleen lymphocytes were cultured in RPMI 1640 (Gibco) supplemented with 10% FBS (Gibco), penicillin (100 U/ml), streptomycin (100 mg/ml), 1 mM sodium pyruvate (Gibco), 55 μM 2-mercaptoethanol (Gibco), and 2 mM L-glutamine. All of these cell lines were cultured at 37 °C with humidified 5% CO_2_. A Mycoalert Mycoplasma Detection Kit (Lonza) was routinely used to monitor mycoplasma contamination.

### Mice

All mice were subjected to specific-pathogen-free conditions (temperature: 21–25 °C; humidity: 30-70%; dark/light cycle: 12 h/12 h) in the State Key Laboratory of Biotherapy (Sichuan University). The mice were sacrificed when the tumor volume reached 1600 mm^3^ or when they were in poor condition and expected to die soon. All animal experiments were approved by the Institutional Animal Care and Use Committee of Sichuan University (Chengdu, Sichuan, China). C57BL/6 (Beijing Vital River Laboratory Animal Technology Co., Ltd.), TLR2^−/−^, TLR4^−/−^, TLR9^−/−^, STING^−/−^, ASC^−/−^, NLRP3^−/−^ and OT-1 mice (Jackson Laboratories) (female, 6–8 weeks old and ~20 g weight) were used.

### Construction of OVA-MITO- and TRP2-MITO-expressing cell lines

We synthesized a cDNA fragment containing sequences encoding the first 32 amino acids of the mouse OTC followed by the full-length sequence encoding OVA (GeneBank: AAB59956.1) or TRP2 amino acids 1-472 (GeneBank: 13190) or mCherry (GeneBank: UFQ89828.1). After digestion by EcoR1 and XhoI restriction enzymes, the purified OTC-OVA/OTC-mCherry fragment was inserted between the EcoR1 and XhoI sites of the pCDH plasmid. Then, we engineered pLV105-TRP2 using the same approach. The constructed plasmids with the packaging vector psPAX2 and the VSV-G encoding plasmid pMD2. G (Addgene) were transiently transfected into HEK293T cells together as previously described [59]. To generate a B16-F10 cell line stably expressing OTC-OVA (OTC-TRP2 and OTC-mCherry), lentiviral particles were transduced into B16-F10 cells, and the clones were selected with puromycin dihydrochloride (2 ng/ml).

### Mitochondria lysate preparation

Cells were collected and resuspended in PBS and washed twice with PBS. A mitochondrial isolation kit (Sigma–Aldrich, 0000104130) was used for the isolation of mitochondria according to the manufacturer’s instructions. In brief, the cells were resuspended in mitochondrial isolation buffer and homogenized with a Dounce homogenizer. Nuclei and cell debris were removed by centrifugation at 600 × *g* for 10 min, and the supernatant containing mitochondria was collected. Then, the mitochondria were concentrated by centrifugation at 3500 × *g* for 10 min. After washing once with PBS, the mitochondria were concentrated by centrifugation at 11,000 × *g* for 10 min. Purified mitochondria were isolated using Percoll density gradient centrifugation [60]. Next, the pellet of purified mitochondria was resuspended in endotoxin-free PBS (Solarbio). A BCA protein assay kit (Thermo Scientific) was used to determine the mitochondrial concentration. All the experiments in this study were performed with freshly isolated mitochondria.

### RNA isolation and real-time quantitative PCR

The Cell Total RNA Isolation Kit (FOREGENE, No. RE-03113) was used for RNA extraction according to the manufacturer’s instructions. cDNA for downstream applications was synthesized using iScript reverse transcriptase (Bio-Rad). Then, cDNA was combined with the gene-specific primers SYBR Master Mix PCR Power SYBR Green (Bio-Rad, 1725124). Real-time quantitative PCR (RT‒qPCR) was performed using the CFX Connect real-time PCR system (Bio-Rad). The PCR products were electrophoresed on 1.5% agarose and visualized using ethidium bromide fluorescence.

### Western blot

Western blot analysis was performed according to a standard protocol. After removing the supernatants, the cells were lysed with RIPA buffer (Beyotime Biotechnology, P0045) in the presence of protease inhibitor cocktail (Med Chem Express, HY-K0010) and phosphatase inhibitor cocktail II (Med Chem Express, HY-K0022) and boiled for 10 min. Then, the proteins were loaded (10 μl/lane), resolved by 12.5% SDS‒PAGE and electroblotted onto PVDF membranes (Millipore). After blocking with 5% (weight/vol) skim milk in Tris-buffered saline–0.1% Tween-20 for 1 h (TBST) at room temperature (RT), PVDF membranes were incubated with the primary antibody at 4 °C overnight. Then, PVDF membranes were washed three times with TBST and subsequently incubated with the secondary antibody in TBST-BSA for 1 h at RT. We replaced the WB blocking buffer with containing primary antibodies (anti-TRP2 mAb, anti-anti-VDAC1 mAb, and anti-Histone H3 mAb) and incubated them overnight at 4 °C. A Supersignal West Pico kit (Thermo Fisher Scientific) was used to detect HRP on PVDF membranes.

### Immunofluorescence staining

Cells were cultured overnight (5 × 10^3^ cells/well) in 24-well plates placed with glass coverslips. Next, the supernatant of the culture medium was aspirated, and the cells were fixed with 4% paraformaldehyde for 30 min, followed by permeabilization with 0.4% Triton X-100. The cells were blocked with 5% fetal bovine serum and washed three times with PBS. The cells were incubated with the indicated primary antibodies, including HSP60 (Abcam, ab46798), OVA (Abcam, ab181688), TRP2 (Biorbyt, orb227952), and LMP2 (Hangzhou Huaan Biotechnology Co., Ltd, ET7107-24), and were then labeled with Goat anti-Rabbit IgG (Heavy Chain) Recombinant Superclonal™ Secondary Antibody, Alexa Fluor® 488 conjugate (Invitrogen, A27034). Finally, the fluorescent signals of the cells were analyzed using a Zeiss LSM 710 confocal microscope. Of note, cells incubated with FITC anti-mouse CD11c antibody (Biolegend, 117306) were not repeatedly labeled with Alexa Flour secondary antibodies.

### Mito-vaccine treatments in vivo

To evaluate the prophylactic effect of the Mito-vaccine, 6-week-old C57BL/6 or TLR2^−/−^ mice were subcutaneously (s.c.) injected (the left flanks of the mice) with 100 μl PBS containing 5 μg soluble OVA (Sigma‒Aldrich, vac-pova-100), 50 μg Mito plus 5 μg soluble OVA, 50 μg Mito or 50 μg OVA-MITO on day 0, 14 and 28. One week after the last immunization, mice were s.c. inoculated with 1×10^6^ E.G7-OVA or 2 × 10^5^ B16-OVA tumor cells on the right flank. For the B16-F10 models, C57BL/6 mice were s.c. injected (the left flanks of the mice) with 100 μl PBS containing 5 μg soluble TRP2, 50 μg Mito or 50 μg TRP2-MITO on day 0, 14 and 28. One week after the last injection, mice were s.c. inoculated with or without 2 × 10^5^ B16-F10 tumor cells on the right flank. One week after tumor inoculation, tumor volume was measured every two days using an electronic caliper and calculated according to the formula (length × width^2^ × 0.52).

To assess the therapeutic effect of the Mito-vaccine, the mice were s.c. inoculated with 1 × 10^6^ E.G7-OVA or 2 × 10^5^ B16-OVA tumor cells on day 0 and then vaccinated three times with 5 μg soluble OVA, 50 μg Mito plus 5 μg soluble OVA or 50 μg OVA-MITO on day 3, 10 and 17. For the B16-F10 models, the mice were s.c. inoculated with 2 × 10^5^ B16-F10 tumor cells on day 0 followed by three inoculations with 100 μl PBS, 50 μg Mito or 50 μg TRP2-MITO on day 3, 10 and 17. One week after the inoculation of tumor cells, tumor volume was measured every two days using an electronic caliper and calculated according to the formula (length × width^2^ × 0.52).

To further investigate the therapeutic effect of the Mito-vaccine in combination with PD-1 antibodies, 6-week-old C57BL/6 mice were s.c. inoculated with 2 × 10^5^ B16-F10 tumor cells on day 0. Then, the mice were vaccinated by s.c. injecting 5 μg soluble TRP2, 50 μg TRP2-MITO or 50 μg Mito on day 3, 10 and 17. Three days after tumor inoculation, mice were intraperitoneally (i.p.) administered 10 mg/kg anti-PD-1 antibodies or isotype-matched control antibodies every 5 days. One week after tumor inoculation, tumor volume was measured every two days using an electronic caliper and calculated according to the formula (length × width^2^ × 0.52).

### Tumor microenvironment

To analyze immune cells infiltrating the tumor microenvironment, C57BL/6 mice were sacrificed on day 14 after incubation with B16-OVA, and the tumor tissues were isolated and digested with collagenase type IV (Gibco, USA) at 37 °C for 30 min. The cells were washed three times with PBS and incubated for 30 min at 4 °C with the following antibodies: PerCP-Cyanine 5.5-conjugated anti-mouse CD3 (Biolegend, 100218), BV421-conjugated anti-mouse Gr1 (Biolegend, 108445), FITC-conjugated anti-mouse CD4 (Biolegend, 100405), PE-conjugated anti-mouse CD107a (Biolegend, 121612), PE-conjugated anti-mouse F4/80 (Biolegend, 123110), APC-conjugated anti-mouse CD8a (Biolegend, 100712), APC-conjugated anti-mouse CD206 (Biolegend, 141708), FITC-conjugated anti-mouse CD45 (Biolegend, 157214), PE-Cy7-conjugated anti-mouse CD11c (Biolegend, 117318), and FITC-conjugated anti-mouse CD11b (Biolegend, 101206). Samples were acquired using a NovoCyte flow cytometer (ACEA Biosciences), and data were collected and analyzed using NovoExpress software.

### In vivo memory T-cell response

Immunized C57BL/6 mice (6–8 weeks) were sacrificed on day 7 after the last injection. The live and dead cells were detected by using a LIVE/DEAD® Fixable Near-IR Dead Cell Stain Kit (Invitrogen, 2277713). The splenic lymphocytes were harvested and then incubated with the following antibodies: FITC-conjugated anti-mouse CD62L (Biolegend, 104406), PerCP-Cyanine5.5-conjugated anti-mouse CD69 (Biolegend, 104522), APC-conjugated anti-mouse CD8 (Biolegend, 100712), BV421-conjugated anti-mouse CD4 (Biolegend, 116023), and BV510-conjugated anti-mouse CD44 (Biolegend, 103044). Samples were acquired and analyzed by a NovoCyte flow cytometer (ACEA Biosciences) with NovoExpress software.

### Analysis of DC mobilization and activation in vivo

To analyze DC mobilization and activation in vivo, ten C57BL/6 mice (*n* = 5 each group) were intradermally injected with PBS, Mito (50 µg). 3h, 6h and 18 h later, the mice were sacrificed, and single-cell suspensions were prepared from the skin and right draining lymph nodes (popliteal lymph nodes and inguinal lymph nodes). The cells were then incubated for 30 min at 4 °C with a combination of fluorochrome-conjugated antibodies against the following surface markers: PerCP-Cyanine5.5-conjugated anti-mouse CD45 (Biolegend, 103132), BV421-conjugated anti-mouse CD11c (Biolegend, 117343), BV650-conjugated anti-mouse MHC II (Biolegend, 107641), PE-conjugated anti-mouse CD8a (Biolegend, 162304), APC-conjugated anti-mouse CD103 (Biolegend, 121414), FITC-conjugated anti-mouse CD11b (Biolegend, 101206), PE-Cy7-conjugated anti-mouse B220 (Biolegend, 103222), and BV421-conjugated anti-mouse CD197 (Biolegend, 120120). Samples were collected using a NovoCyte flow cytometer (ACEA Biosciences).

### DC maturation in vitro

Bone marrow-derived DCs (BMDCs) from wild-type or TLR2^−/−^ mice and TLR4^−/−^, TLR9^−/−^, STING^−/−^, ASC^−/−^, and NLRP3^−/−^ mice were prepared as described previously [61] and cultured in RPMI 1640 (Gibco) supplemented with 10% FBS (Gibco), penicillin (100 U/ml), streptomycin (100 mg/ml) with IL-4 (10 ng/ml) (PeproTech, AF-214-14) and GM-CSF (20 ng/ml) (PeproTech, AF-315-03). The activation and maturation of BMDCs were detected after incubation with OVA-MITO or mitochondria lysate for 24 h. Briefly, the culture supernatants were collected, and the levels of IL-6, TNF-α and IL-12p70 in DC-conditioned medium were measured using ELISA kits (Invitrogen, 88-7064-88, 88-7324-88, 88-7121-88). The cells were collected and stained with FITC-conjugated anti-mouse CD40 (Biolegend, 553790), PE-conjugated anti-mouse CD86 (Biolegend, 105008), APC-conjugated anti-mouse CD11c (Biolegend, 117310), BV421-conjugated anti-mouse CD80 (Biolegend, 104725), and PE-Cy7-conjugated anti-mouse MHC II antibodies (Biolegend, 107630). Samples were collected using a NovoCyte flow cytometer (ACEA Biosciences).

### Mitochondrial staining and DC uptake

To label the mitochondria, the MitoTracker Red CMXRos dye (Thermo Fisher, I34154) stock solution was diluted to a working concentration of 50 nM by supplementing the medium. B16-F10 cells were then incubated in MitoTracker Red CMXRos for 20 min at 37 °C. Then, the labeling solution was removed, and the cells were washed twice with the culture medium. Next, the mitochondrial isolation procedure was performed on ice in the dark as described above.

BMDCs were incubated with the prelabeled mitochondria. After incubation for 3–6 h, the DCs were washed and fixed with 4% paraformaldehyde, followed by staining with hamster primary anti-CD11c antibody (Biolegend, 117306) for 30 min. FITC-conjugated rabbit anti-hamster IgG secondary antibodies were then added and incubated for 1 h at 37 °C (Invitrogen). The cell nuclei were labeled with DAPI (Beyotime, P0131) according to the manufacturer’s instructions. The DCs were then mounted and analyzed under an LMS 710 (Carl Zeiss) confocal microscope or analyzed by a NovoCyte flow cytometer (ACEA Biosciences).

### Inhibition and activation of BMDCs by TLR2 inhibitors

BMDCs from wild-type mice were pretreated with C29 (100 μM) (Med Chem Express, 363600-92-4), a specific inhibitor of TLR2, for 2 h and then treated with or without mitochondria (10 μg/ml) for 24 h. The culture supernatants were collected to measure the levels of IL-6, TNF-α and IL-12p70. The cells were incubated with the following antibodies: FITC-conjugated anti-mouse CD40 (Biolegend, 553790), PE-conjugated anti-mouse CD86 (Biolegend, 105008), APC-conjugated anti-mouse CD11c (Biolegend, 117310), BV421-conjugated anti-mouse CD80 (Biolegend, 104725), and PE-Cy7-conjugated anti-mouse MHC II (Biolegend, 107630).

### DC-based immunotherapy in vivo

To assess the therapeutic effect of mitochondria-pulsed DC-based immunotherapy in vivo, mitochondria (10 μg/ml) from OVA-MITO-expressing B16-F10 cells or Vehicle-MITO-expressing B16-F10 cells were cocultured with 1 × 10^6^ C57BL/6 mouse-derived DCs per 10 cm cell culture dish, followed by LPS (10 μg/ml) to stimulate BMDCs overnight. Next, 6-week-old C57BL/6 mice were s.c. inoculated with 1 × 10^6^ E.G7-OVA or 2 × 10^5^ B16-OVA tumor cells. After three days, 1 × 10^6^ mitochondria-pulsed DCs were s.c. injected at weekly intervals for 3 weeks.

For the B16-F10 models, mitochondria (10 μg/ml) from TRP2-MITO-expressing B16-F10 cells or Vehicle-MITO-expressing B16-F10 cells were cocultured with BMDCs, which were then stimulated with LPS. The C57BL/6 mice were s.c. inoculated with 2 × 10^5^ B16-F10 tumor cells and treated with mitochondria-pulsed DCs in the same injection schedule. One week after tumor inoculation, tumor volume was measured every two days using an electronic caliper and calculated by the formula (length × width^2^ × 0.52).

### In vivo T-cell response

C57BL/6 mice were s.c. injected with 100 μl PBS containing 5 μg soluble OVA, 50 μg mito plus 5 μg soluble OVA (OVA/mito) or 50 μg OVA-MITO at weekly intervals for 3 weeks. For the B16-F10 models, C57BL/6 mice (6–8 weeks) were s.c. injected with 100 μl PBS containing 50 μg Mito or 50 μg TRP2-MITO at weekly intervals for 3 weeks. One week after the last injection, all mice were sacrificed, and the splenic lymphocytes were harvested and purified using mouse lymphocyte separation medium (Dakewe). Isolated splenocytes were directly used for staining with TRP2-tetramers (Biolegend, CPM-1-0448) or OVA-tetramers (MBL, TS-5001-1C). After the incubation, FITC anti-mouse CD8 antibody (Biolegend, 553031) was added and incubated. This was followed by detection with a NovoCyte flow cytometer (ACEA Biosciences). Then, the freshly isolated lymphocytes were restimulated with OVA_257–264_ (SIINFEKL) peptide (Invivogen, vac-sin), OVA_323–339_ (ISQAVHAAHAEINEAGR) peptide (10 μg/ml) (Invivogen, vac-isq) or TRP2_180-188_ (SVYDFFVWL) (Shanghai Toshisun Biology and Technology Co., Ltd.) for 72 h. Brefeldin A (Biolegend, 420601) was added to the cell cultures 4 h before harvesting the cells. The cells were collected and stained using the LIVE/DEAD™ Fixable Violet Dead Cell Stain Kit (Invitrogen) for live cells. Then, the cells were stained with FITC anti-mouse CD8 antibody (Biolegend, 553031). Subsequently, the cells were fixed and permeabilized with a Fixation/Permeabilization Solution Kit (BD Biosciences, 554714) and incubated with PE-conjugated anti-mouse IFN-γ (Biolegend, 505808) or APC-conjugated anti-mouse granzyme B (Biolegend, 515406) overnight at 4 °C. Samples were acquired using a NovoCyte flow cytometer (ACEA Biosciences). In addition, the T-cell-conditioned medium was collected for the measurement of the levels of IFN-γ using the IFN gamma “Femto-HS” High Sensitivity Mouse ELISA Kit (Invitrogen, 88-8314-88).

### Cytotoxic T lymphocyte (CTL) assay

To obtain effector T cells, splenic lymphocytes from immunized mice were isolated and stimulated with SIINFEKL peptide (10 μg/ml) in the presence of interleukin-2 (20 ng/ml) (Sigma–Aldrich) for 72 h. B16-OVA cells used as the targets were seeded in 96-well plates (5 × 10^3^ cells/well) and cocultured with different groups of effector T cells at different effector-to-target (E:T) ratios. After incubation for 6 h at 37 °C, the cell culture supernatants were collected, and lactate dehydrogenase activity was measured using a CytoTox96 Non-Radioactive Cytotoxic Assay kit (Promega, G1781, G1782) according to the manufacturer’s instructions.

### Adoptive transfer and immunization in vivo

C57BL/6 mice were s.c. injected with 5 μg soluble OVA, 50 μg mito plus 5 μg soluble OVA or 50 μg OVA-MITO following a three-dose immunization regimen spaced 7 days apart. The immunized mice were sacrificed on day 21 after the first vaccination for the collection of splenic lymphocytes, and a total of 1 × 10^7^ lymphocytes in 100 μl PBS were intravenously (i.v.) injected into C57BL/6 mice. Recipient mice were then s.c. injected with 2 × 10^5^ B16-OVA cells after the reception of lymphocytes. The tumor sizes were measured and calculated as previously described.

### The proliferation assay of OT-I CD8^+^ T lymphocytes

BMDCs derived from wild-type or TLR2^−/−^ mice were cultured for 6 days, and the cells were then stimulated with OVA (1 μg/ml) or OVA-MITO (10 μg/ml) for 18 h. Then, the cells were collected and resuspended in RPMI 1640 for further studies.

The CD8^+^ T lymphocytes from the spleen of OT-1 mice were prepared through CD8 magnetic bead negative selection using a Mouse CD8^+^ T-Cell Isolation Kit (Stem Cell). Next, the CD8^+^ T lymphocytes were resuspended in 2.5 μM CFSE labeling solution and then incubated at 37 °C for 15 min. Subsequently, serum-containing medium was added and incubated for 5 min. After washing three times with PBS, the OT-1 CD8^+^ T lymphocytes were cocultured with OVA- or OVA-MITO-treated BMDCs at a 5:1 ratio for 48 or 72 h. The number of CD8^+^ T lymphocytes was counted under a white light microscope after 48 h of incubation. After 72 h of coculture, the nonadherent cells were harvested, and the CFSE fluorescence value of T cells was analyzed by flow cytometry.

### Preparation and characterization of liposomes

To prepare the liposomes, cardiolipin was synthesized by the solvent injection method as previously described [62]. Briefly, cardiolipin (Sigma‒Aldrich, C0563-100MG) and cholesterol were dissolved in absolute ethanol at 65 °C (mass ratio = 2:1). This mixed solution was added dropwise through a 1 ml syringe into a stirred aqueous phase at 65 °C. The volume of this mixed solution was reduced to 2/3 by a heating magnetic stirrer at 65 °C to remove the absolute ethanol. TEM (H-600, Hitachi, Japan) and DLS (Malvern Zetasizer Nano ZS) were used to determine the size distribution and morphology of cardiolipin liposomes.

### Therapeutic effect of TPR2-MITO combined with cardiolipin

To evaluate the therapeutic effect of TPR2-MITO in combination with cardiolipin, C57BL/6 mice were s.c. inoculated with 2 × 10^5^ B16-F10 tumor cells on day 0. Then, mice were s.c. injected with 50 μg TRP2-MITO on day 3, day 10, and day 17. In addition, 3 days after tumor inoculation, mice were intraperitoneally (i.p.) administered 5 mg/kg cardiolipin every 3 days. Tumor volume was measured on day 7 after inoculation and calculated as described above.

### Crls1 siRNA transfection and knockdown

The following RNAi sequences were used: si*Crls1* #1 (rat, 5′- CGAACACTAGCTAAGTACT-3′); si*Crls1* #2 (rat, 5′-CAGCTCCAGTTTTCAATTA -3′); si*Crls1* #3 (rat, 5′- AACATTCATCAGCAAGGTAAATACA-3′). Stably transfected *TRP2* cells were seeded in six-well plates (2 × 10^3^ cells/well) and transfected with siRNA oligonucleotides specific for cardiolipin or negative control siRNA using Lipofectamine 3000 Transfection Reagent (Invitrogen, L3000-015). The next day, the cells were replenished with fresh complete media and harvested 48 h later to measure the cardiolipin RNA and cardiolipin content in the cells. The cardiolipin content was analyzed by a Cardiolipin Assay Kit (Abcam, ab241036) according to the manufacturer’s instructions.

### Crls1 siRNA mito-vaccine treatment in vivo

Control and Crls1 siRNA-transfected cells were treated with Lipofectamine 3000 Transfection Reagent as described above and were collected 48 h after transfection. Next, mitochondria were extracted from the siRNA-transfected cells. In addition, an in vivo experiment using a Crls1 siRNA mito-vaccine (si*Crls1*) and a control mito-vaccine (si*Scramble*) was performed. Briefly, 6-week-old C57BL/6 mice were s.c. inoculated with 2 × 10^5^ B16-F10 tumor cells on day 0. Then, mice were s.c. injected with 100 μl PBS containing 50 μg si*Crls1* or 50 μg si*Scramble* on day 3, 10, and 17. One week after tumor cell inoculation, tumor volume was measured and calculated as described above.

### Safety evaluation of the mito-vaccine in healthy mice

To assess the safety of different Mito-vaccines in vivo, the appearance and weight of vaccinated mice were monitored every day. The serum samples of the vaccinated mice were collected for the analysis of biochemical parameters. Specifically, the indicators of serum biochemical parameters, including aspartate aminotransferase (AST), alanine aminotransferase (ALT), blood urea nitrogen (BUN), creatinine (CREA), and alkaline phosphatase (ALP) enzymes, were detected by COBAS (Roche Diagnostics). The Anti-mitochondrial antibody content was analyzed by Anti-mitochondrial antibody (AMA) ELISA Kit (MYBIOSOURCE, MBS262592) according to the manufacturer’s instructions.

### Statistical analysis

All statistical analyses were performed using GraphPad Prism software v8.02 (ns, not significant; *P < 0.05; ** *P* < 0.01; *** *P* < 0.001; and **** *P* < 0.0001). The data are presented as the mean ± standard error. Data were analyzed using unpaired *t* test or one*-*way ANOVA. The log-rank (Mantel-Cox) test was used to evaluate the significant difference in survival analysis.

## Supplementary information

Supplementary Information

Original images of gels and western blots

## Data Availability

All data are available in the main text or the supplementary materials.
