# Supplementary material for: Engineered mitochondria exert potent antitumor immunity as a cancer vaccine platform
Source: Cell Mol Immunol. 2024 Aug 20;21(11):1251–65. doi: 10.1038/s41423-024-01203-4 (PMC11528120; doi:10.1038/s41423-024-01203-4)
Supplement: Supplementary file 1 — Supplementary Information [file 41423_2024_1203_MOESM1_ESM.pdf]

Supplementary Information for

Engineered mitochondria exert potent antitumor immunity as a cancer vaccine platform

Correspondence to: xiaweiwei@scu.edu.cn (Editorial corresponding author)

Includes:

Supplemental Fig 1-5

Supplementary Information

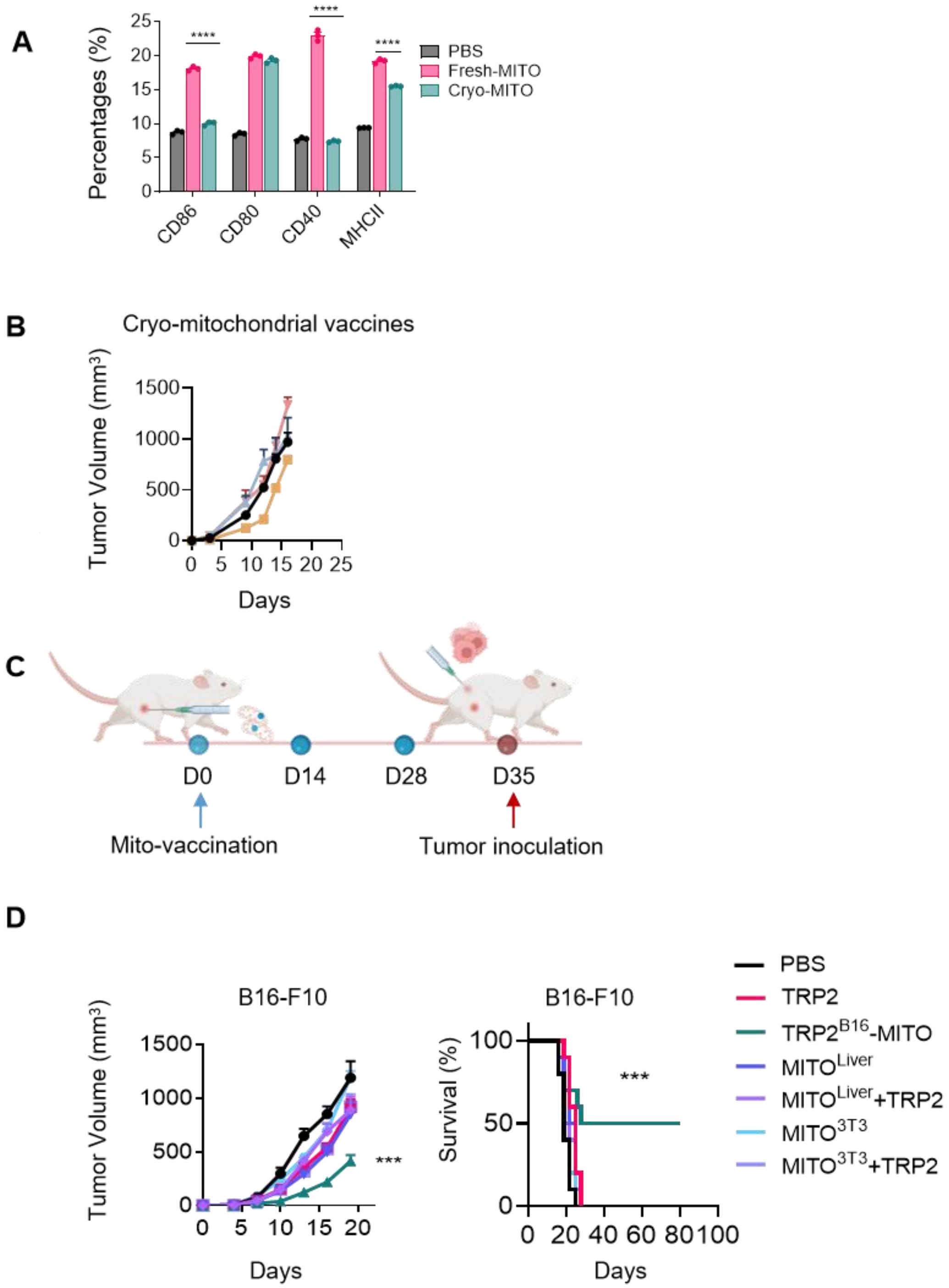

Supplementary Fig. 1. Fresh-TRP2-MITO derived from tumor cells can efficiently inhibited tumor growth.

A, Flow cytometric analysis of CD86, CD80, CD40, and MHC II expression in BMDCs treated with Fresh-MITO or Cryo-MITO (10 μg/ml) *in vitro* for 24 h. **B,** B16-F10 tumor-bearing WT and TLR2$^{-/-}$ mice received the indicated therapeutic treatments on the opposite frank (50 μg

cryo-Mito or 50 μg cryo-TRP2-MITO on day 3, 10 and 17). Tumor volumes measured at the indicated time points are shown. (n = 8 in the WT Mito group and $TLR2^{-/-}$ TRP2-MITO; n = 9 in the other groups). **C,** Schematic representation of the prophylactic tumor vaccines (TRP2, $TRP2^{B16}$-MITO, $MITO^{Liver}$, $MITO^{Liver}$+TRP2, $MITO^{3T3}$ or $MITO^{3T3}$+TRP2) for immunotherapy in mice. **D,** B16-F10-derived tumor-bearing mice received the indicated prophylactic treatments (5 μg soluble TRP2, 50 μg $TRP2^{B16}$-MITO, 50 μg $MITO^{Liver}$, 50 μg $MITO^{Liver}$ plus 5 μg soluble TRP2, 50 μg $MITO^{3T3}$ plus 5 μg soluble TRP2, or 50 μg $MITO^{3T3}$ on day 0, 14 and 28). Tumor volumes and mouse survival measured at the indicated time points are shown. (n = 10). Tumor volumes and mouse survival measured at the indicated time points are shown. Data are presented as the mean values ± SEMs. One-way ANOVA was conducted for analysis of tumor volumes, and the log rank (Mantel–Cox) test was used for survival; ***$P < 0.001$, and ****$P < 0.0001$.

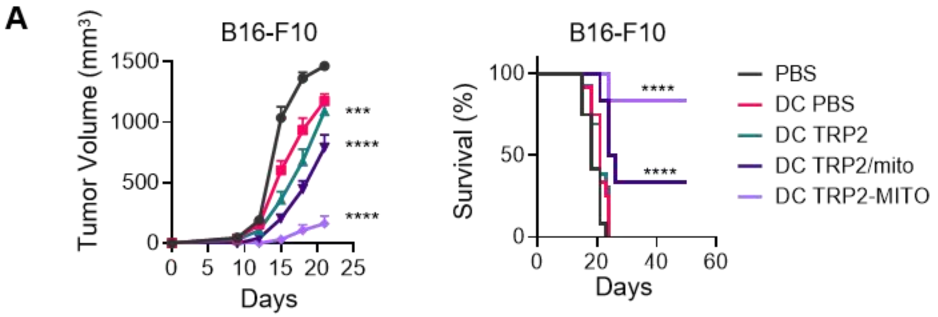

Supplementary Fig. 2. TRP2-MITO can be utilized in the form of a DC cell-based cancer vaccine and elicits a potent therapeutic antitumor effect.

A, B16-F10 tumor-bearing mice received the indicated therapeutic treatments (1×10^6 DCs were preincubated with PBS, TRP2, TRP2/mito and TRP2-MITO overnight and injected on day 3, 10 and 17). Tumor volumes and mouse survival measured at the indicated time points are shown. (n = 12) Data are presented as the mean values ± SEMs. One-way ANOVA was conducted for analysis of tumor volumes, and the log rank (Mantel–Cox) test was used for survival; ***P < 0.001, and ****P < 0.0001.

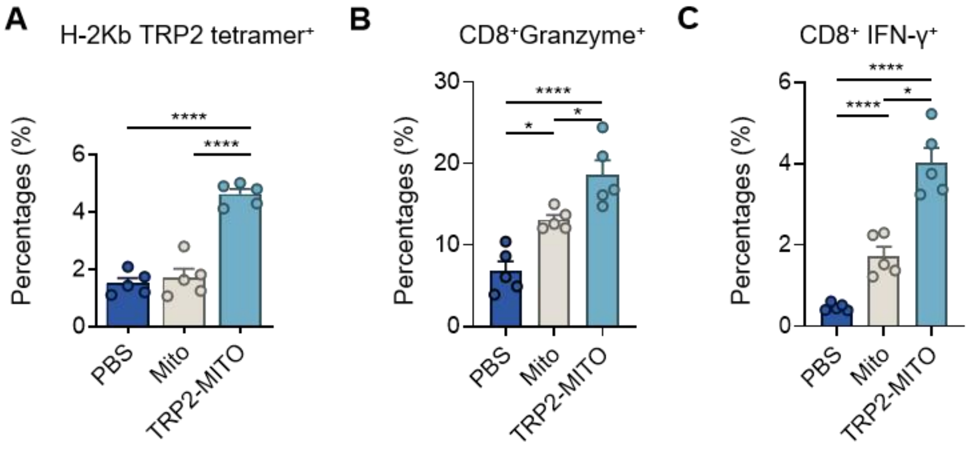

Supplementary Fig. 3. The TRP2-MITO vaccine elicits T-cell immunity in the tumor microenvironment.

A, Splenocytes from mice that had received the indicated treatment (50 μg Mito or 50 μg TRP2-MITO immunized on day 0, 14 and 28) were isolated and subsequently cultured *in vitro* with $TRP2_{180\text{-}188}$ (SVYDFFVWL) peptide for 72 h. Flow cytometric analysis of TRP2 tetramer$^+$ CD8$^+$ T cells. (n = 3). **B-C,** Flow cytometric analysis of CD8$^+$granzyme$^+$ (B) and CD8$^+$ IFN-γ$^+$ (C) T cells after the same treatments as (A). (n = 5). Data are presented as the mean values ± SEMs. One-way ANOVA was conducted in (A) to (D); *P < 0.05, and ****P < 0.0001.

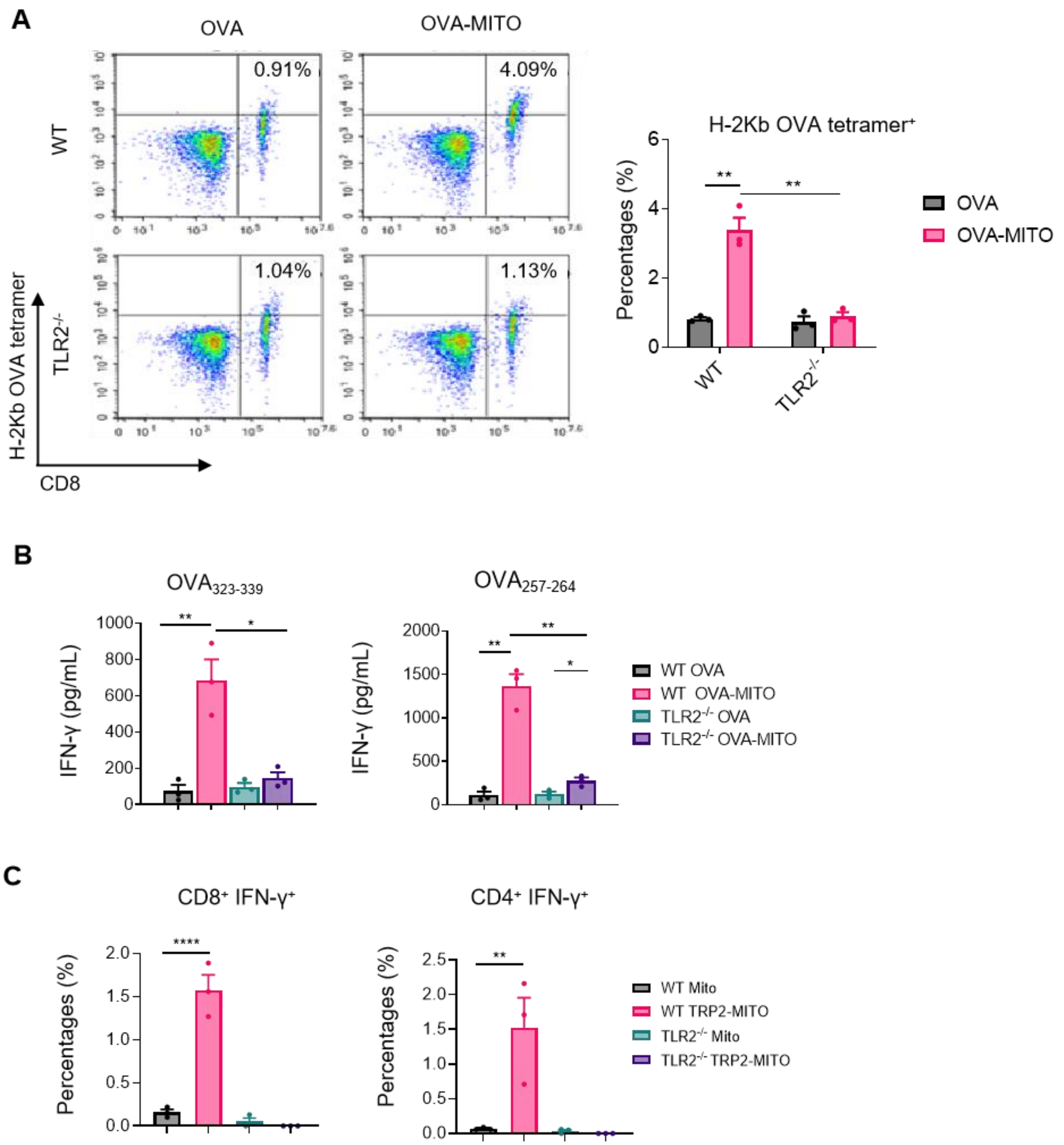

Supplementary Fig. 4. Mitochondria vaccines cannot stimulate cross-priming of a primary $CD8^+$ T response in $TLR2^{-/-}$ mice.

A, Splenocytes from WT and $TLR2^{-/-}$ mice that had received the indicated treatment (5 μg soluble OVA or 50 μg OVA-MITO on day 0, 14 and 28) were isolated and subsequently cultured *in vitro* with $OVA_{257\text{-}264}$ peptide (10 μg/mL) for 72 h. Flow cytometric analysis of OVA tetramer$^+$ $CD8^+$ T cells. (n = 3). **B,** Splenocytes from WT and $TLR2^{-/-}$ mice that had received the indicated treatment (5 μg soluble OVA or 50 μg OVA-MITO on day 0, 14 and 28) were isolated and subsequently cultured *in vitro* with $OVA_{323\text{-}339}$ or $OVA_{257\text{-}264}$ peptides (10 μg/mL) for 72 h. IFN-γ

concentration in the supernatant of the cells. (n = 3). **C,** Splenic lymphocytes from mice that had received the indicated treatment (50 μg Mito or 50 μg TRP2-MITO on day 0, 14 and 28) were isolated and subsequently cultured *in vitro* with $TRP2_{180-188}$ (SVYDFFVWL) peptide (10 μg/mL) for 72 h. Flow cytometric analysis of IFN-γ^{+} $CD8^{+}$ T cells and IFN-γ^{+} $CD4^{+}$ T cells. (n = 3). Data are presented as the mean values ± SEMs. One-way ANOVA was conducted in (A) to (C); *$P < 0.05$, **$P < 0.01$, and ****$P < 0.0001$.

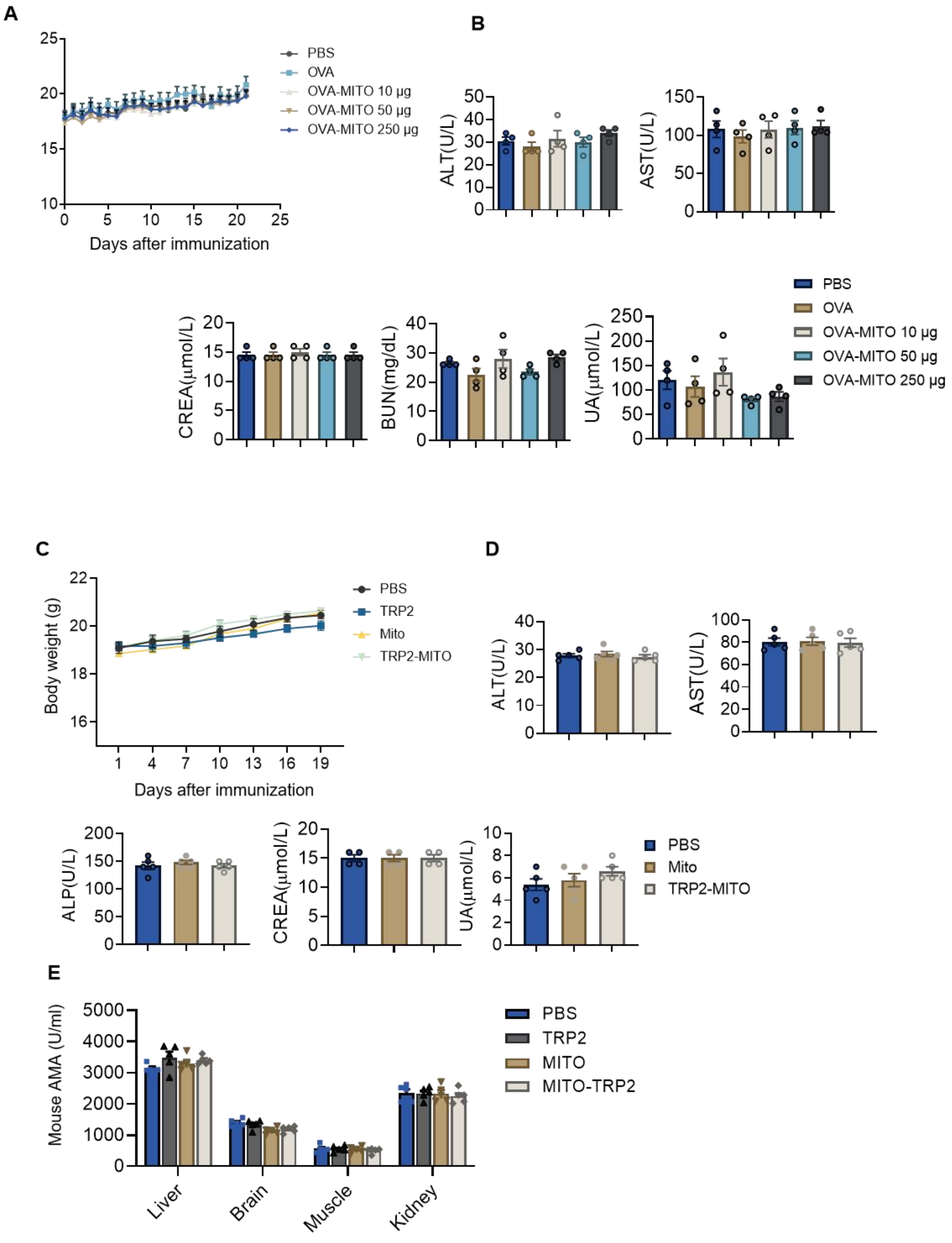

Supplementary Fig. 5. Safety analysis of engineered mitochondria vaccines.

A, C57BL/6 mice received OVA (5 µg) or the indicated concentrations (10, 50, 250 µg) of OVA-MITO on day 0, 14 and 28. The body weight of mice measured at the indicated time points

is shown. (n = 6). **B,** C57BL/6 mice received the same treatments as (A). Serum biochemical analysis measured at day 31 is shown. (n = 4). **C,** C57BL/6 mice received the indicated treatments (5 μg TRP2, 50 μg Mito, 50 μg TRP2-MITO on day 0, 14 and 28). The body weight of mice measured at the indicated time points is shown. (n = 12). **D,** C57BL/6 mice received the same treatments as (C). **E,** Anti-mitochondrial antibody analysis measured at day 31 is shown. (n = 5). Serum biochemical analysis measured at day 31 is shown. (n = 5). Data are presented as the mean values ± SEMs.
