# Supplementary figures and images for: Engineered mitochondria exert potent antitumor immunity as a cancer vaccine platform
Source: Cell Mol Immunol. 2024 Aug 20;21(11):1251–65. doi: 10.1038/s41423-024-01203-4 (PMC11528120; doi:10.1038/s41423-024-01203-4)

# Original images of gels and western blots

Fig.1b Up

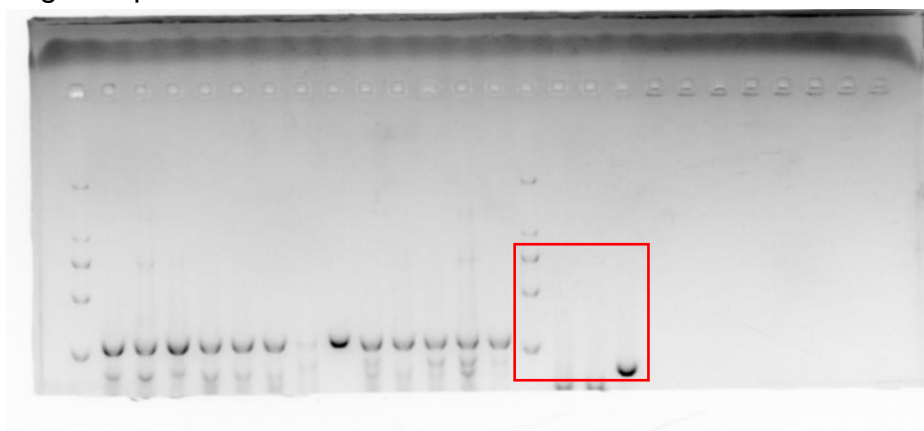

Fig.1b Bottom

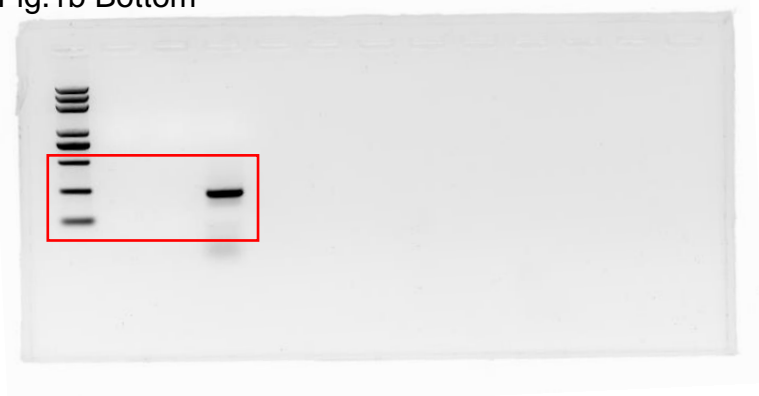

Fig.1c Left

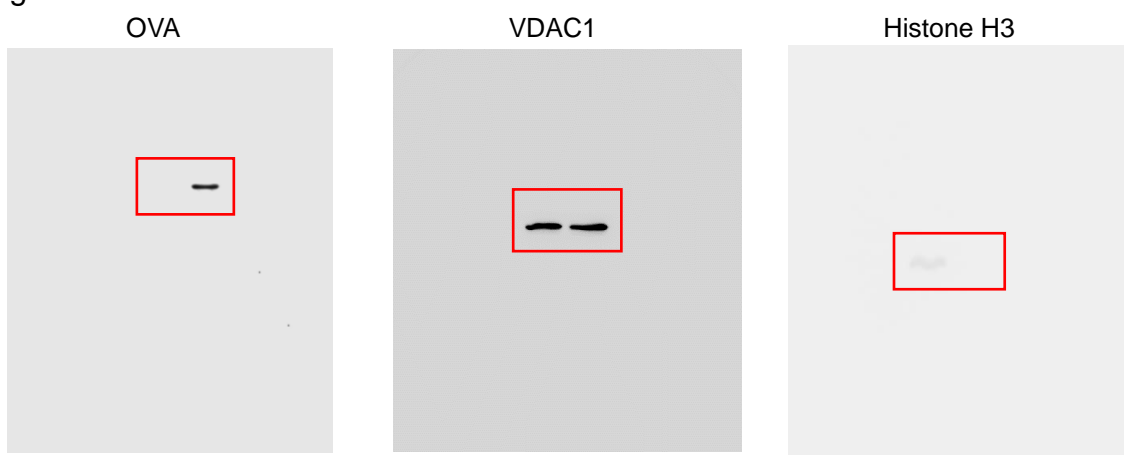

Fig.1c Right

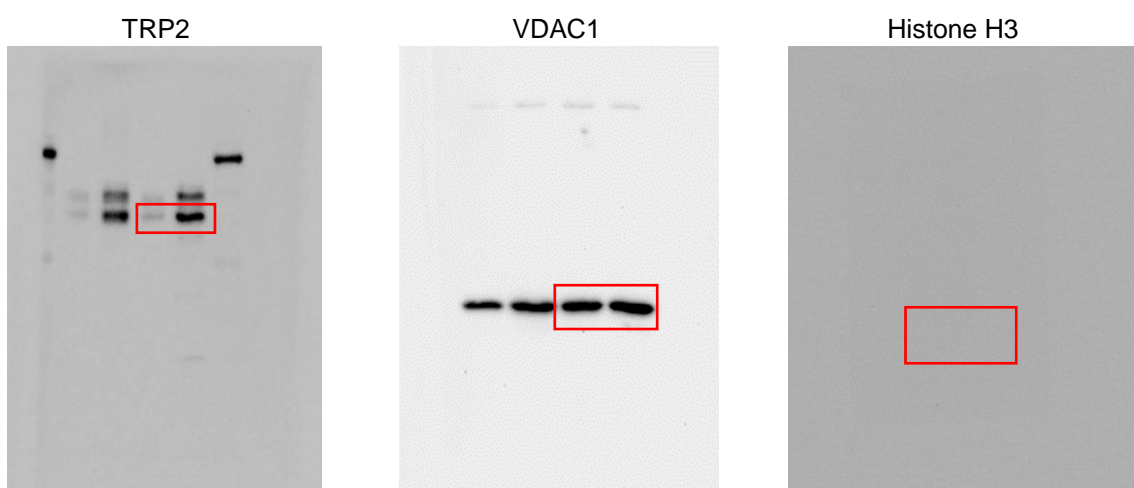

Supplement: Supplementary file 2 — Original images of gels and western blots [file 41423_2024_1203_MOESM2_ESM.pdf]
